# Replicative Instability Drives Cancer Progression

**DOI:** 10.3390/biom12111570

**Published:** 2022-10-26

**Authors:** Benjamin B. Morris, Jason P. Smith, Qi Zhang, Zhijie Jiang, Oliver A. Hampton, Michelle L. Churchman, Susanne M. Arnold, Dwight H. Owen, Jhanelle E. Gray, Patrick M. Dillon, Hatem H. Soliman, Daniel G. Stover, Howard Colman, Arnab Chakravarti, Kenneth H. Shain, Ariosto S. Silva, John L. Villano, Michael A. Vogelbaum, Virginia F. Borges, Wallace L. Akerley, Ryan D. Gentzler, Richard D. Hall, Cindy B. Matsen, C. M. Ulrich, Andrew R. Post, David A. Nix, Eric A. Singer, James M. Larner, Peter Todd Stukenberg, David R. Jones, Marty W. Mayo

**Affiliations:** 1 Department of Biochemistry and Molecular Genetics, University of Virginia, Charlottesville, VA 22908, USA; 2 Department of Pathology, University of Virginia, Charlottesville, VA 22908, USA; 3 Center for Public Health Genomics, University of Virginia, Charlottesville, VA 22908, USA; 4 M2Gen, Tampa, FL 34667, USA; 5 Division of Medical Oncology, Department of Internal Medicine, Markey Cancer Center, Lexington, KY 40536, USA; 6 Division of Medical Oncology, Department of Internal Medicine, The Ohio State University Comprehensive Cancer Center, Columbus, OH 43210, USA; 7 Department of Thoracic Oncology, H. Lee Moffitt Cancer Center and Research Institute, Tampa, FL 33612, USA; 8 Division of Hematology/Oncology, Department of Internal Medicine, University of Virginia Comprehensive Cancer Center, Charlottesville, VA 22908, USA; 9 Department of Breast Oncology, H. Lee Moffitt Cancer Center and Research Institute, Tampa, FL 33612, USA; 10 Huntsman Cancer Institute and Department of Neurosurgery, University of Utah, Salt Lake City, UT 84112, USA; 11 Department of Radiation Oncology, The Ohio State University Comprehensive Cancer Center, Columbus, OH 43210, USA; 12 Department of Malignant Hematology, H. Lee Moffitt Cancer Center and Research Institute, Tampa, FL 33612, USA; 13 Department of Cancer Physiology, H. Lee Moffitt Cancer Center and Research Institute, Tampa, FL 33612, USA; 14 Department of NeuroOncology, H. Lee Moffitt Cancer Center, Tampa, FL 33612, USA; 15 Division of Medical Oncology, University of Colorado Comprehensive Cancer Center, Aurora, CO 80045, USA; 16 Department of Medical Oncology, Department of Internal Medicine, Huntsman Cancer Institute, Salt Lake City, UT 84112, USA; 17 Department of Surgery, Huntsman Cancer Institute, University of Utah, Salt Lake City, UT 84112, USA; 18 Huntsman Cancer Institute and Department of Population Health Sciences, University of Utah, Salt Lake City, UT 84112, USA; 19 Department of Biomedical Informatics and Huntsman Cancer Institute, University of Utah, Salt Lake City, UT 84112, USA; 20 Department of Oncological Sciences, Huntsman Cancer Institute, Salt Lake City, UT 84112, USA; 21 Section of Urologic Oncology, Rutgers Cancer Institute of New Jersey, New Brunswick, NJ 08901, USA; 22 Department of Radiation Oncology, University of Virginia Comprehensive Cancer Center, Charlottesville, VA 22908, USA; 23 Department of Thoracic Surgery, Memorial Sloan-Kettering Cancer Center, New York, NY 10065, USA

**Keywords:** replicative instability (RIN), cancer progression, metastasis, MYBL2, single-strand break repair, translesion synthesis

## Abstract

In the past decade, defective DNA repair has been increasingly linked with cancer progression. Human tumors with markers of defective DNA repair and increased replication stress exhibit genomic instability and poor survival rates across tumor types. Seminal studies have demonstrated that genomic instability develops following inactivation of BRCA1, BRCA2, or BRCA-related genes. However, it is recognized that many tumors exhibit genomic instability but lack BRCA inactivation. We sought to identify a pan-cancer mechanism that underpins genomic instability and cancer progression in BRCA-wildtype tumors. **Methods:** Using multi-omics data from two independent consortia, we analyzed data from dozens of tumor types to identify patient cohorts characterized by poor outcomes, genomic instability, and wildtype BRCA genes. We developed several novel metrics to identify the genetic underpinnings of genomic instability in tumors with wildtype BRCA. Associated clinical data was mined to analyze patient responses to standard of care therapies and potential differences in metastatic dissemination. **Results:** Systematic analysis of the DNA repair landscape revealed that defective single-strand break repair, translesion synthesis, and non-homologous end-joining effectors drive genomic instability in tumors with wildtype BRCA and BRCA-related genes. Importantly, we find that loss of these effectors promotes replication stress, therapy resistance, and increased primary carcinoma to brain metastasis. **Conclusions:** Our results have defined a new pan-cancer class of tumors characterized by replicative instability (RIN). RIN is defined by the accumulation of intra-chromosomal, gene-level gain and loss events at replication stress sensitive (RSS) genome sites. We find that RIN accelerates cancer progression by driving copy number alterations and transcriptional program rewiring that promote tumor evolution. Clinically, we find that RIN drives therapy resistance and distant metastases across multiple tumor types.

## 1. Background

Large-scale sequencing efforts have enabled the discovery of genetic events that drive cancer development [1,2,3,4,5]. Analysis of sequencing data has helped establish molecular classifiers through which tumors can be grouped by mutations, copy number changes, or fusions. Careful study of genetic drivers has greatly expanded our understanding of how cancers develop. Despite this knowledge, primary tumors with similar genetic backgrounds often have heterogeneous outcomes, suggesting that additional factors beyond initial oncogenic events influence patient outcomes. This is especially true when considering cancer progression events like therapy resistance and metastasis.

Decades of research has revealed processes dysregulated by cancers, summarized as hallmarks of cancer [6,7]. Of these hallmarks, genomic instability has been linked to progressive disease across cancers [8,9]. Familial studies and genome analyses have revealed that genomic instability can develop following inactivation of BRCA1, BRCA2, or BRCA-related genes [10]. As a result, tumors rely on error-prone repair and accumulate mutations and chromosomal alterations [10]. Clinically, tumors with defective BRCA can be targeted using PARP inhibitors and chemotherapies [11]. However, it is recognized that genomic instability is observed in tumors that lack BRCA inactivation [12]. Importantly, these tumors respond poorly to PARP inhibition, chemotherapy, and irradiation [12]. Defining the genetic underpinnings of tumors with genomic instability and wildtype BRCA is a significant unmet need in cancer research.

Previous work from our group revealed that elevated expression of the transcription factor MYB proto-oncogene like 2 (MYBL2) identified lung adenocarcinomas with genomic instability and wildtype BRCA [13]. In this manuscript, we sought to identify a pan-cancer mechanism that underpins genomic instability and cancer progression in BRCA-wildtype tumors. In this study, we provide evidence that elevated *MYBL2* expression is a robust marker of poor patient outcomes across tumor types and genotypes. Importantly, this *MYBL2* High cohort is defined by genomic instability despite containing wildtype BRCA. Analysis of the DNA repair landscape revealed that the genetic basis of *MYBL2* High disease are heterozygous losses of single-strand break repair, translesion synthesis, or non-homologous end-joining effectors. These genetic lesions subject *MYBL2* High tumors to significant replication stress. Functional clustering of replication stress-sensitive sites revealed that elevated replication stress promotes copy number alterations that rewire transcriptional programs and impact hallmarks of cancer master regulators. Clinically, this phenotype identifies patients at risk for poor outcomes when treated with chemotherapy and irradiation. Furthermore, our results demonstrate that *MYBL2* expression stratifies patient risk for distant metastases, especially to the brain. Our data defines a new pan-cancer class of tumors driven by replicative instability (RIN), unifying seemingly disparate cancers. Moreover, these results describe how RIN accelerates cancer progression by impacting several hallmarks of cancer.

## 2. Methods

### 2.1. TCGA Pan-Cancer Analysis

Thirty-two tumor types curated by the TCGA and others were analyzed [14]. Where multiple TCGA studies were available, we analyzed PanCancer studies. RNA-sequencing samples were stratified into *MYBL2* High and *MYBL2* Low cohorts using a quartile method; the top 25% of samples expressing *MYBL2* were called *MYBL2* High and the bottom 25% *MYBL2* Low.

### 2.2. Survival Analyses

The Kaplan-Meier estimator was used to estimate time-to-event distributions for OS, DSS, and PFS comparing MYBL2 High and MYBL2 Low quartiles. The log-rank test was used to test for significant differences between distributions using a two-sided test. OS denotes the time from initial diagnosis until death. DSS is the time from diagnosis until the time of death; patients who died from other causes were not included. PFS reflects the time from diagnosis until progression or death. For all survival analyses, patients who did not experience an event or were lost to follow-up were censored at time of last contact. Kaplan-Meier survival analyses were conducted using survival and survminer packages [15].

### 2.3. DNA Repair Pathway WE Score

For each pathway, we identified comprehensive lists of pathway effectors through extensive literature review [16,17,18,19,20,21,22,23,24,25]. Effectors were scored based on essentiality to pathway function (Essentiality Scaling Factor (ESF): 3 = essential effector, 2 = important effector/potentially compensable, 1 = accessory effector). Repair pathway genes and their associated ESF values are listed in Supplementary Table S3. The final WE formula for each pathway is a scaled average where gene log2 fold change (Log2FC) values are multiplied by an ESF, summed, and divided by the number of pathway genes.

$$WE=\frac{\left(Gene\;A\;Log2FC\right)\left(ESF\right)+\left(Gene\;B\;Log2FC\right)\left(ESF\right)+\left(Gene\;C\;Log2FC\right)\left(ESF\right)+\ldots }{\#\;Pathway\;genes}$$

Correlations between WE values were calculated and visualized using stats and corrplot R packages [26].

### 2.4. Catalogue of Somatic Mutations in Cancer (COSMIC) v3.2 Single Base Substitution (SBS) Analysis

COSMIC SBS v3.2 signatures were generated using deConstructSigs [27]. For TCGA cohorts, the TCGA public MAF file was used to generate trinucleotide mutation contexts. For ORIEN cohorts, individual sample vcf files were used to calculate trinucleotide mutation contexts. The final deConstructSigs output was computed using the trinucleotide context matrix and the COSMIC v3.2 SBS signature matrix downloaded from (https://cancer.sanger.ac.uk/signatures/downloads/). Eighteen of the 78 SBS mutational signatures capturing likely artifacts were excluded. Statistical significance between average signature weights across samples was assessed using two-sided Student’s *t*-tests.

### 2.5. RS Score 

Eight gene ontology (GO) terms were identified that capture key processes involved in replication stress responses (GO:0031570, GO:0000076, GO:006260, GO:0031261, GO:004311, GO:0031297, GO:0031298, GO:0071932). Genes were pooled and redundant entries removed to generate a final list (n = 205). The RS score is the sum of gene log2 mRNA expression values, divided by the number of genes in the RS response gene list. Differences in medians were assessed for statistical significance using Wilcoxon signed rank tests [28].

$$RS\;Score=\frac{\left(Gene\;1\;expression\right)+\left(Gene\;2\;expression\right)+\left(Gene\;3\;expression\right)+\ldots }{205}$$

### 2.6. MPS 

The MPS score is the difference between somatic mutation location and the gene start, divided by gene length.

$$MPS=\frac{Mutation\;position\;\left(bp\right)-Gene\;start\;\left(bp\right)}{Gene\;length\;\left(bp\right)}\;$$

Mutation locations were obtained from the TCGA public MAF file. Gene start and end positions were obtained from the Ensembl genome browser (www.ensembl.org). Differences in MPS densities were assessed for statistical significance using Kolmogorov-Smirnov tests.

### 2.7. RSS Site Alteration Analysis

RSS sites were identified by Barlow et al. [29] and Macheret et al. [30]. ERFS were obtained from Table S1 “Ordered_List_of_ERFS_Hot_Spots” [29]. Genes were mapped to human gene identifiers using nichenetr [31]. MiDAS sites were obtained from Supplementary Table S1 [30]. Sites were filtered to include MiDAS sites attributable to one or two genes. Final ERFS and MiDAS sites were merged to identify overlapping genes. This merged identified 20 genes called ERFS but recently defined as MiDAS. These genes were removed from the ERFS list and analyzed in the MiDAS list. Copy number and mutation frequencies were plotted using ggplot2 and ggridges R packages [28]. Differences in medians were assessed for statistical significance using Wilcoxon signed rank tests [28].

### 2.8. RSS Site Functional Analysis

ERFS and MiDAS genes were combined and analyzed for biologic processes using WebGestalt’s over-representation analysis feature. Thirteen functional clusters were defined and genes were binned into clusters following literature review (Supplementary Table S5). Single-cell RNA expression data from the Human Protein Atlas was used to ensure genes were expressed in tissues relevant to our tumor cohorts. This final gene list with functional cluster annotation was merged with differential expression RNA-seq tables. Combined copy number and RNA-seq files were analyzed and genes with significant copy number and transcriptional differences were identified (Supplementary Table S6). Circular packing diagrams were drawn using ggraph and igraph R packages [32,33].

### 2.9. Tumor Microenvironment Analysis

Immune cell estimates were generated using the ConsensusTME package [34]. Tumor type infiltration estimates were calculated separately using tumor-specific gene sets and a single-sample Gene Set Enrichment Analysis (ssGSEA) method [34]. MDSC estimates, Dysfunction, and Exclusion Scores were downloaded from TIDE [35].

### 2.10. ORIEN Therapy Response

Data from 25 tumor types provided by ORIEN were analyzed. For samples with RNA-seq data, we manually reviewed treatment records to identify patients treated with chemotherapeutics or irradiation. For treatment-specific cohorts, samples were stratified into *MYBL2* High and *MYBL2* Low cohorts using a quartile method. Kaplan-Meier analyses were performed as described above.

### 2.11. FUSED

FUSion Error-prone repair Detection (FUSED) was developed to map the origin of RNA-seq detected fusions. Tool rules were determined through literature review [36]. FUSED is available at https://github.com/databio/FUSED.

### 2.12. ORIEN Metastatic Dissemination

ORIEN medical records were manually reviewed to identify sites of metastatic disease. Metastatic routes were plotted using circlize [37]. Medical records were used to calculate the time from diagnosis to metastatic dissemination. Times to metastasis were plotted using the swimplot R package [38]. Differences in time to metastasis data were assessed using Wilcoxon signed rank tests [28]. Brain metastasis risk was calculated by multiplying the number of patients that develop metastatic disease by the number of patients with brain metastases. This fraction was multiplied by 100% to generate final risk percentages.

### 2.13. Statistical Analyses

Statistical tests for all analyses are indicated in figure legends. For boxplots, data are graphed as minimum, 1st-quartile, median, 3rd-quartile, and maximum. *p*, *q* < 0.05 were considered statistically significant.

## 3. Results

### 3.1. Pan-Cancer Analysis Identifies MYBL2 Expression as a Robust Marker of Poor Patient Outcomes

**Figure 1:**
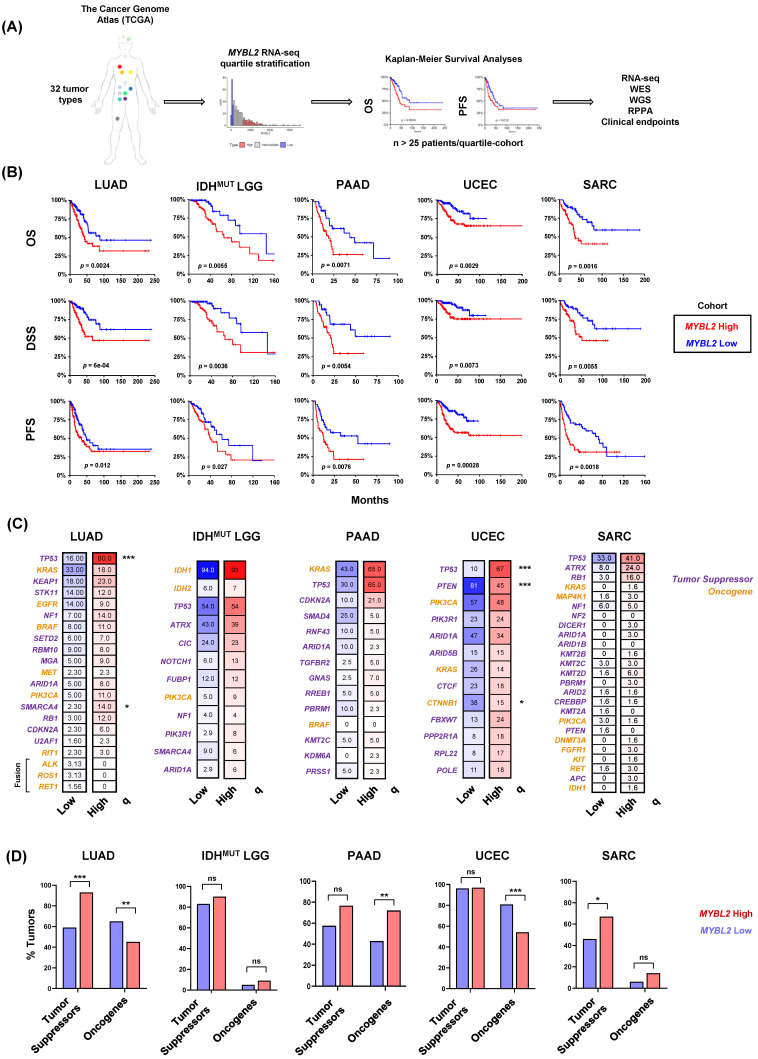
Elevated *MYBL2* mRNA expression identifies patients with poor outcomes across multiple tumor types and genotypes. (**A**) Pan-cancer analysis overview. (**B**) Kaplan-Meier analyses demonstrate that *MYBL2* expression is robustly prognostic across multiple tumor types for OS, DSS, and PFS outcomes. Log-rank test *p*-values are displayed. (**C**) *MYBL2* High tumors develop across common cancer genetic driver backgrounds. Percentages reflect the percent of tumors with gene specific alterations. Statistical significance mapping represents Benjamini-Hochberg corrected *q* values, *q* < 0.05 *, *q* < 0.001 ***. (**D**) Individual tumors show different patterns of tumor suppressor inactivation and oncogene activation with respect to *MYBL2* High and *MYBL2* Low disease. IDH^MUT^ LGG tumor suppressor and oncogene status were mapped excluding founding IDH mutations. One-sided Fisher’s exact test, *p* < 0.05 *, *p* < 0.01 **, ns: not significant. LUAD: lung adenocarcinoma. IDH^MUT^ LGG: IDH-mutant lower grade glioma. PAAD: pancreatic adenocarcinoma. UCEC: uterine corpus endometrial carcinoma. SARC: sarcoma.

To test if *MYBL2* expression identified patients with poor outcomes and progressive disease across tumor types, we analyzed 32 studies curated by The Cancer Genome Atlas (TCGA) and other groups [14]. For each study, samples were stratified based on *MYBL2* mRNA expression using a quartile approach (Figure 1A). To be included in further analyses, *MYBL2* expression had to identify patients with significantly inferior overall survival (OS) and progression-free survival (PFS) outcomes. Kaplan-Meier analyses confirmed that *MYBL2* expression was a robust marker of poor patient outcomes in multiple tumor types, including lung adenocarcinoma (LUAD, n = 128 patients/arm), isocitrate dehydrogenase (IDH)-mutant lower grade glioma (IDH^MUT^ LGG, n = 104 patients/arm), pancreatic adenocarcinoma (PAAD, n = 44 patients/arm), uterine corpus endometrial carcinoma (UCEC, n = 132 patients/arm), and sarcoma (SARC, n = 63 patients/arm) (Figure 1B, Supplementary Tables S1 and S2). Across these tumor types, patients with *MYBL2* High disease had significantly worse OS, disease-specific survival (DSS), and PFS outcomes compared to *MYBL2* Low patients.

*MYBL2* High and Low tumors were profiled for tumor-specific genetic driver events as defined previously (Figure 1C) [1,2,3,4,5]. This analysis demonstrated that *MYBL2* High tumors develop across genotypes, with few statistically significant enrichments for individual driver alterations. Notable exceptions include enrichments for TP53 mutations in LUAD and UCEC. Given this lack of enrichment, driver genes were binned into broad tumor suppressor and oncogene categories to test for general enrichment patterns (Figure 1D). This analysis also failed to identify a clear pattern, indicating that additional steps beyond known driver mutations are required to generate *MYBL2* High tumors.

### 3.2. MYBL2 High Tumors Are Characterized by Genomic Instability despite Containing Wildtype BRCA

**Figure 2:**
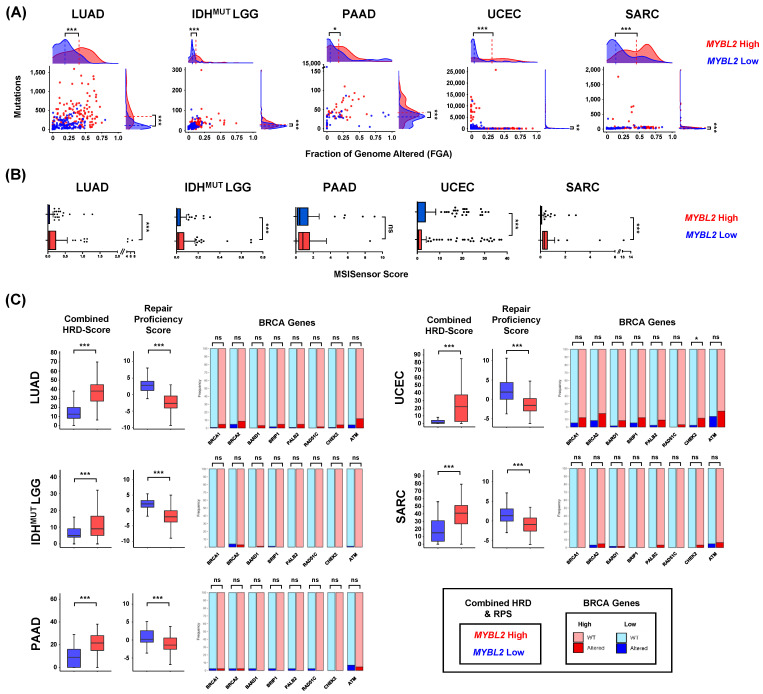
*MYBL2* High tumors exhibit genomic instability despite containing wildtype BRCA genes. (**A**) *MYBL2* High tumors have significantly greater somatic mutation burdens and fraction of the genome (FGA) altered. (**B**) *MYBL2* High tumors have elevated microsatellite instability scores. (**C**) *MYBL2* High tumors exhibit inefficient homologous recombination despite containing wildtype BRCA genes. (**A**–**C**) Statistical significance was assessed using Wilcoxon signed rank tests. *p* < 0.05 *, *p* < 0.01 **, *p* < 0.001 ***. (**C**) Enrichments for inactivating alterations in BRCA genes were tested using one-sided Fisher’s exact tests. Significance is mapped using Benjamini-Hochberg corrected *q* values. *q* < 0.05 *, *q* < 0.001 ***; ns, not significant.

To characterize similarities of *MYBL2* High disease, we analyzed DNA damage metrics provided by the TCGA PanCancer consortium [14,39]. We found *MYBL2* High tumors universally had elevated mutation burden and greater fractions of the genome altered (FGA) (Figure 2A). All *MYBL2* High tumor cohorts exhibited greater levels of microsatellite instability (MSI) (Figure 2B). It should be noted that only a small number of samples reach the threshold required to be deemed ‘MSI-High’ (MSISensor ≥ 10), most of which are UCECs [40]. Regardless, separating tumors based on *MYBL2* mRNA expression consistently identified tumors with varying degrees of elevated MSI. Taken together, these data demonstrate that genomic instability is a hallmark of *MYBL2* High disease.

Studies have shown that a common cause of genomic instability is a loss of homologous recombination (HR) repair [10]. To analyze the status of HR repair, we analyzed combined homologous recombination deficiency (combined HRD) scores and repair proficiency scores (RPS) [39,41]. Combined HRD scores quantify genomic scars as they reflect the sum of chromosomal alterations impacting telomeres, loss of heterozygosity events, and large-scale transitions [39]. Tumors with high combined HRD scores exhibit elevated genomic instability. The RPS is an RNA-based metric that reflects expression of double-strand break repair effectors [41]. Low RPS values indicate tumors with dysfunctional HR [41]. *MYBL2* High tumors, regardless of tumor type, exhibited significantly elevated combined HRD scores and decreased RPS scores, compared to *MYBL2* Low tumors (Figure 2C). Taken together, two orthogonal metrics indicate that *MYBL2* High tumors have inefficient HR.

Inefficient HR has been linked to inactivating mutations or deletions in BRCA genes [10]. Given this, we profiled *MYBL2* High and Low tumors for somatic mutations or homozygous deletions in BRCA genes (Figure 2C). Surprisingly, mutations and deletions in BRCA genes were rare in *MYBL2* High tumors. More importantly, these alterations were not significantly enriched when comparing *MYBL2* High and Low cohorts (Figure 2C). One exception was an enrichment for CHEK2 alterations in *MYBL2* High UCEC. Careful inspection of Figure 2C reveals increased inactivating alterations in our LUAD and UCEC cohorts. This is likely because LUAD is linked to carcinogen exposure and several *MYBL2* High UCEC tumors carry POLE mutations. These results indicate that *MYBL2* High tumors fall into the clinically relevant category of tumors with genomic instability and wildtype BRCA.

### 3.3. Heterozygous Loss of Repair Effectors Underly Defective DNA Repair in MYBL2 High Tumors

**Figure 3:**
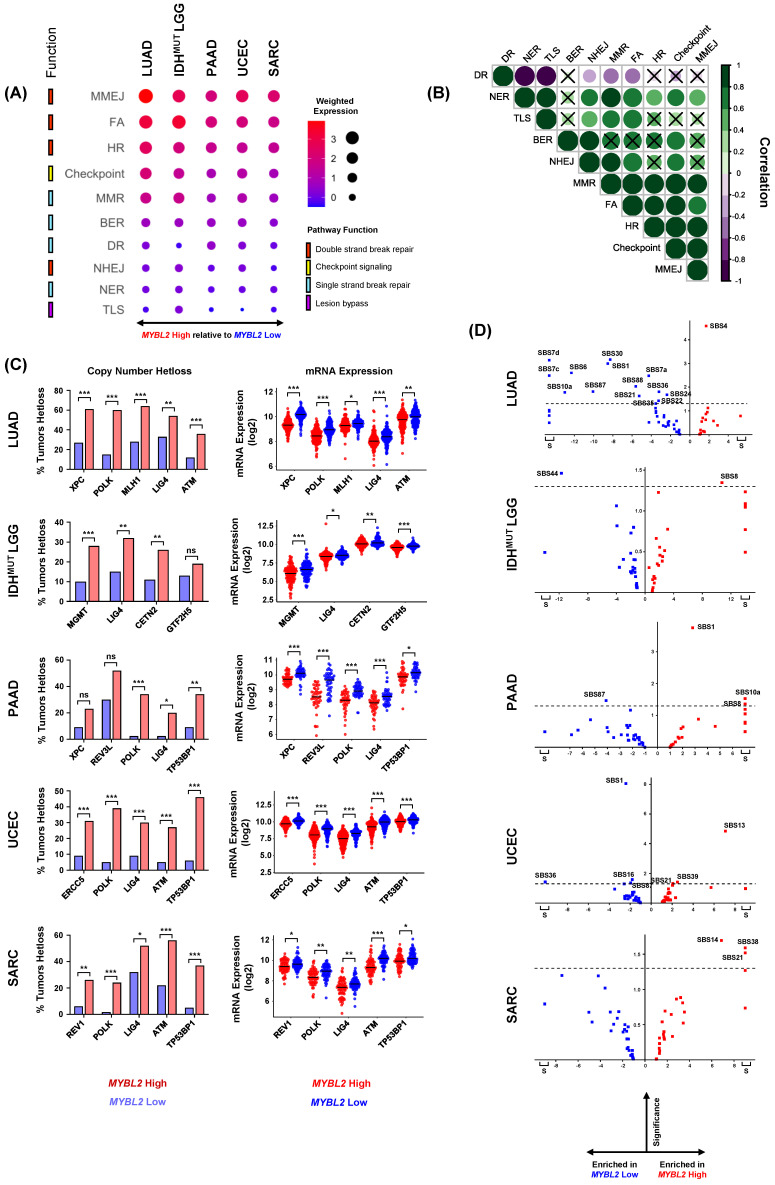
Heterozygous losses impacting key DNA repair effectors are enriched in *MYBL2* High tumors. (**A**) Weighted expression scores reveal an imbalance in DNA repair pathway regulation. (**B**) Observed differences in WE scores are highly correlated across different cancer types. Correlations with x marks indicate correlations that are not statistically significant (Pearson). (**C**) Heterozygous losses in genes encoding key single-strand break repair, TLS, and NHEJ effectors are highly enriched in *MYBL2* High tumors. One-sided Fisher’s exact test, *p* < 0.05, *; *p* < 0.01, **; *p* < 0.001, ***. Heterozygous loss events are highly correlated with decreased expression of repair effectors. Benjamini-Hochberg corrected *q*. *q* < 0.05, *; *q* < 0.01, **, *q* < 0.001, ***. (**D**) COSMIC v3.2 SBS analysis reveals heterozygous loss of repair effectors is associated with impaired pathway function. S: Signatures specifically observed only in *MYBL2* High or *MYBL2* Low tumors. Dotted line represents Student’s *t*-test *p* = 0.05.

Given the lack of BRCA inactivation, we characterized the DNA repair landscape in *MYBL2* High tumors in search of the genetic origin of genomic instability. We developed a weighted expression (WE) score to describe how expression of repair pathways is regulated in tumors (2. Methods). This metric was applied to all single-strand break repair pathways (SSBR), double-strand break repair (DSBR) pathways, and checkpoint and lesion-bypass mechanisms (Figure 3A). Analysis of WE pathway scores revealed a striking imbalance between the expression of different pathways. Key DBSR pathways and checkpoint pathways were robustly upregulated across *MYBL2* High tumors while SSBR pathways showed different degrees of downregulation (Figure 3A). Across tumor types, we found that translesion synthesis (TLS), nucleotide excision repair (NER), and non-homologous end-joining (NHEJ) pathways were consistently the most downregulated pathways. Correlation analysis demonstrated patterns observed in Figure 3A were strongly correlated across cancer types (Figure 3B). A notable tumor-specific event was the downregulation of direct repair (DR) observed in *MYBL2* High IDH^MUT^ LGG.

We next asked if downregulated pathway scores were driven by decreased expression of individual effector genes. Close inspection revealed that *MYBL2* High tumors exhibited strong downregulation of individual effectors (Supplementary Table S3). We profiled *MYBL2* High tumors for genetic alterations that could account for this specific dysregulation. Homozygous deletions and inactivating mutations were highly infrequent and could not explain the expression differences observed in Figure 3A. Additional analysis revealed that the driver of pathway dysregulation in *MYBL2* High tumors were specifically enriched heterozygous loss events impacting key repair effectors (Figure 3C). These heterozygous loss events were highly correlated with decreased effector mRNA expression (Figure 3C). Across cancers, we found that heterozygous loss events in XPC (2/5 tumor types), POLK (4/5), LIG4 (5/5), ATM (3/5), and TP53BP1 (3/5) were common in *MYBL2* High tumors. Heterozygous loss of MGMT was specific to *MYBL2* High IDH^MUT^ LGG, fitting with previous reports of DR impairment being a tissue-specific driver of oncogenesis [42].

To assess the functional impact of these heterozygous loss events, we analyzed the Catalog of Somatic Mutations in Cancer (COSMIC) v3.2 single-base substitution (SBS) signature data (Figure 3D) [43]. This analysis identified several signatures that were enriched in *MYBL2* High tumors. SBS8 was over-represented in both *MYBL2* High IDH^MUT^ LGG and PAAD. SBS8 is characterized by increased C>A transversions and has been linked to deficient NER [43]. This fits well given that *MYBL2* High IDH^MUT^ LGG have increased heterozygous losses in NER effectors CETN2 and GTF2H5 (Figure 3C). Also, *MYBL2* High PAAD have significantly increased heterozygous losses affecting both XPC and POLK. XPC and POLK mediate the first and last steps of NER, respectively [16]. SBS21 was over-represented in *MYBL2* High UCEC and SARC. SBS21 captures increased T>C transversions and has been linked with NER defects [43]. Previously, we identified that *MYBL2* High UCEC carried heterozygous losses in ERCC5 and POLK. Similarly, *MYBL2* High SARC have also significantly increased heterozygous losses in POLK. Lastly, signature SBS4 was over-represented in *MYBL2* High LUAD. SBS4 features increased C>A transversions and is the byproduct of tobacco smoke [44]. Importantly, *MYBL2* High LUAD carried heterozygous losses in XPC and POLK which impair cellular ability to repair smoking-induced lesions through NER [16]. This analysis supports the notion that heterozygous losses of repair effectors functionally decrease pathway repair efficiency.

### 3.4. Defective SSBR and TLS Are Linked to Increased Replication Stress and Distinct Genomic Footprints

**Figure 4:**
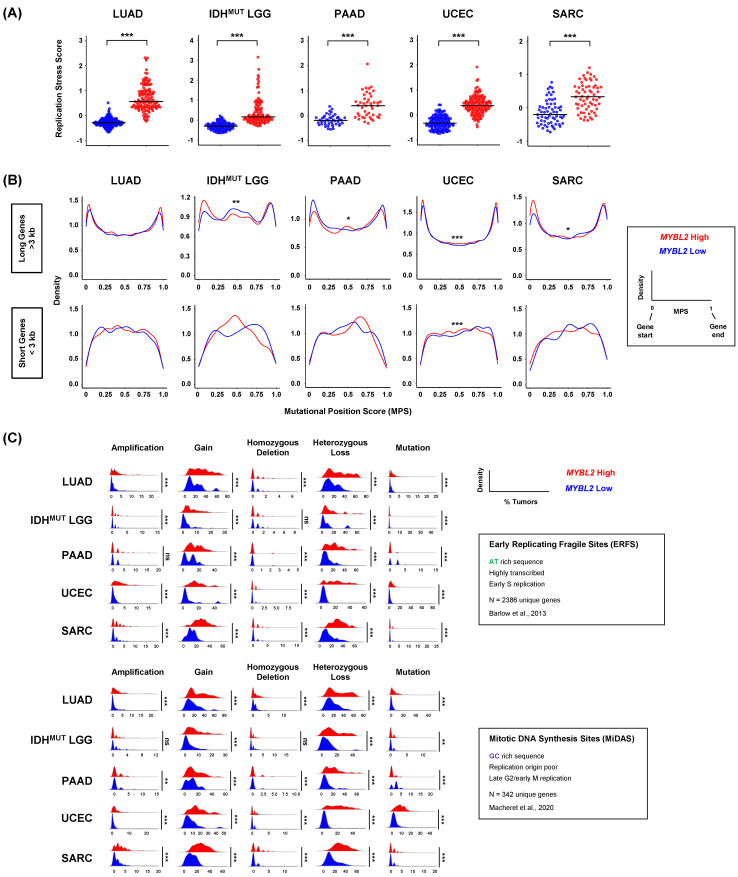
*MYBL2* High tumors exhibit markers of chronic replication stress. (**A**) *MYBL2* High tumors universally demonstrate significantly elevated replication stress scores. Wilcoxon, *p* < 0.001, ***. (**B**) *MYBL2* High tumors experience a shift in intragenic somatic mutation position, relative to *MYBL2* Low tumors. Kolmogorov-Smirnov test, *p* < 0.05, *; *p* < 0.01, **; *p* < 0.001, ***. (**C**) *MYBL2* High tumors acquire significantly greater numbers of alterations at replication stress sensitive genomic sites. Wilcoxon, *p* < 0.01, **; *p* < 0.001, ***. ERFS sites [29], MiDAS sites [30].

SSBR and TLS pathways are essential for safe-guarding replication. Various SSBR pathways help regulate the speed and accuracy of replicative polymerases. TLS represents an essential lesion-bypass mechanism that helps alleviate replication fork stalling when the replicative machinery encounters DNA lesions [17]. Genetic models of defective SSBR and TLS demonstrate genomic instability and elevated replication stress [45]. Cells contain multiple pathways that sense and respond to replication dysregulation [18]. Elevated expression of genes in these pathways are indicative of cells that experience significant replication stress [46]. To investigate if *MYBL2* High tumors experience elevated replication stress, we developed a novel metric called the replication stress score (RS score) (Methods). This metric captures all major pathways involved in sensing replication stress, protecting and processing stalled forks, and the rescue of replication [46]. When comparing *MYBL2* High and Low cohorts, we found that *MYBL2* High tumors universally exhibited elevated RS scores (Figure 4A). This suggests that *MYBL2* High tumors struggle with DNA replication, likely stemming from decreased SSBR and TLS (Figure 3). Based on findings in Figure 4A, we asked if *MYBL2* High tumors accumulate somatic mutations at different locations across intragenic regions. To test this hypothesis, we developed a metric called the mutational position score (MPS) (Methods). This metric allows us to directly compare the spatial location of mutations in individual genes across tumors. Here, MPS values near 0 represent mutations close to gene starts, while values near 1 reflect mutations close to gene ends. MPS values near 0.5 represent mutations accumulating in the middle of gene bodies. When comparing MPS traces, we found *MYBL2* High tumors experience significant shifts in intragenic mutation location (Figure 4B). For this analysis, we subdivided all genes into long genes (>3000 bp) and short genes (<3000 bp). For long genes, *MYBL2* High tumors acquired more mutations near gene starts and gene ends, likely stemming from transcription-replication conflicts. Analysis of short genes showed more pronounced changes, where *MYBL2* High tumors showed increased mutation in gene bodies (Figure 4B). The lack of significance for these patterns likely stems from fewer mutations in short genes, compared to long genes. Collectively, this shift in mutational position is consistent with increased replication stress and impaired SSBR and TLS function seen across *MYBL2* High tumors.

It has long been understood that certain genomic loci are sensitive to replication stress [47]. Recent studies have subdivided these loci into two categories, early replicating fragile sites (ERFS) and mitotic DNA synthesis sites (MiDAS) [29,30]. Genes encoded at these sites are sensitive to replication stress due to their DNA sequence, replication timing, and location in the genome. We hypothesized that *MYBL2* High tumors accumulate more alterations at replication stress sensitive (RSS) sites. Using copy number and WES data, we profiled *MYBL2* High and Low tumors for amplifications, gains, homozygous deletions, heterozygous losses, and mutations impacting ERFS and MiDAS sites (Methods). Across both groups, we found that *MYBL2* High tumors accumulate significantly greater numbers of genetic alterations (Figure 4C). Strikingly, we found that the number of gene-level gains and heterozygous losses dwarfed those of amplifications, homozygous deletions, or mutations. Additionally, previously reported copy number trends associated with replication timing did not correlate with our findings [48]; *MYBL2* High tumors acquired similar numbers of gains and heterozygous losses across both ERFS and MiDAS, with a trend toward more heterozygous losses (Figure 4C). These data are consistent with previous findings where elevated MMEJ activity is coincident with increased loss of heterozygosity [49]. Across tumor types, we observed strong right-handed tailing indicating that many genes are impacted by gains or heterozygous losses in >30–40% of *MYBL2* High tumors (Figure 4C). These data suggest that repeated gene-level gains and heterozygous losses at RSS sites originate from increased replication stress.

### 3.5. Recurrent Copy Number Alterations at RSS Sites Rewire Transcriptional Programs and Impact Hallmark of Cancer Master Regulators

**Figure 5:**
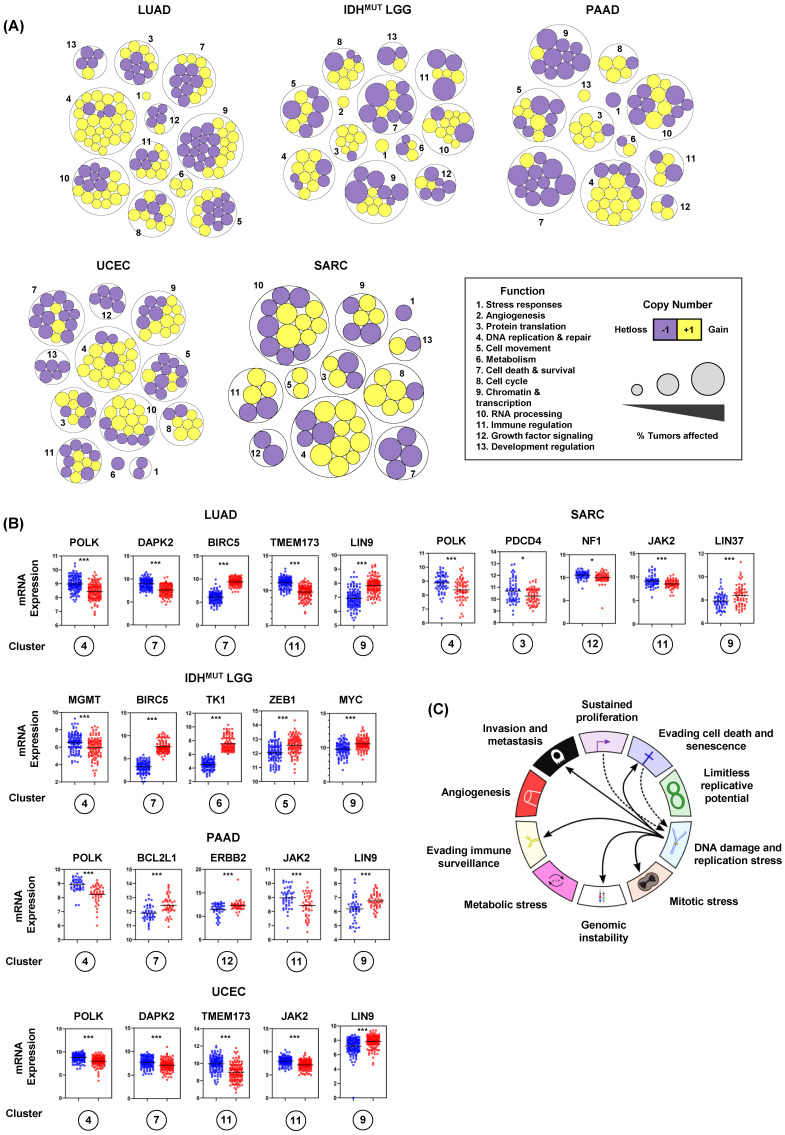
Recurrent copy number alterations at replication stress sensitive sites rewire transcriptional programs and dysregulate master effectors controlling several hallmarks of cancer. (**A**) *MYBL2* High tumors acquire copy number alterations in essential enzymes encoded at replication stress sensitive sites. (**B**) Enriched copy number alterations observed in *MYBL2* High tumors rewire transcriptional programs and dysregulate master effectors controlling several hallmarks of cancer. Statistical significance is mapped according to Benjamini-Hochberg corrected *q* values. *q* < 0.05, *; *q* < 0.001, ***. Circled cluster numbers map to those displayed in (**A**). (**C**) Replication stress dysregulates master effectors controlling several hallmarks of cancer.

After noticing that large numbers of genes were recurrently altered, we examined the function of genes encoded at RSS sites (Methods). Biological process analysis revealed that genes encoded at RSS sites fit into thirteen functional categories (Figure 5A). Importantly, we found that conserved copy number changes significantly impacted gene expression (Figure 5A,B). Across cancers, we found that *MYBL2* High tumors frequently gained copies of genes controlling DNA replication and repair. Similarly, we found recurrent heterozygous losses impacting multiple genes controlling cell survival. While some events were confined to individual tumor types, there was striking conservation of genes altered across *MYBL2* High tumors (Figure 5A, Supplementary Figures S1–S5). Further analysis revealed that copy number alteration and subsequent transcriptional regulation impacted master effectors that regulate several hallmarks of cancer. We observed repeated heterozygous loss and transcriptional downregulation of *TMEM173/STING1*, *DAPK2*, *POLK*, *JAK2*, *NF1*, and *PDCD4* (Figure 5B). Recurrent copy number gains and transcriptional upregulation was observed for *BCL2L1*, *LIN9*, *ZEB1*, *MYC*, *TK1*, *LIN37*, and *ERBB2/HER2* (Figure 5B). These results indicate that increased replication stress, stemming from heterozygous repair effector loss, promotes dysregulation of key master regulators encoded at RSS sites (Figure 5C).

### 3.6. MYBL2 High Tumors Exhibit Immunosuppressive Microenvironments

Given increased dysregulation of key effectors controlling immune regulation, we sought to characterize the immune microenvironment associated with MYBL2 High tumors. As expected, we found that MYBL2 High tumors have significantly elevated neoantigen loads (Supplementary Figure S6A) [50]. Interestingly, we found that MYBL2 High tumors lacked statistically significant differences in CD8+ T-cell infiltration (Supplementary Figure S6B) [34]. However, MYBL2 High tumors universally demonstrated elevated infiltration of myeloid-derived suppressor cells (MDSCs) (Supplementary Figure S6B) [35]. Across tumor types, we also found these tumors were associated with greater Exclusion and decreased Dysfunction scores. Lastly, analysis of tumor hypoxia scores revealed MYBL2 High tumors are significantly hypoxic (Supplementary Figures S6C and S12) [14]. Increased hypoxia scores fit well with increased MDSC infiltration and significantly decreased infiltration of endothelial cells across MYBL2 High tumors (Supplementary Figures S7–S11). These data indicate that MYBL2 High tumors exhibit uniquely dysregulated, immunosuppressive microenvironments.

### 3.7. Elevated MYBL2 Identifies Patients at Risk for Therapy Failure and Distant Metastases

**Figure 6:**
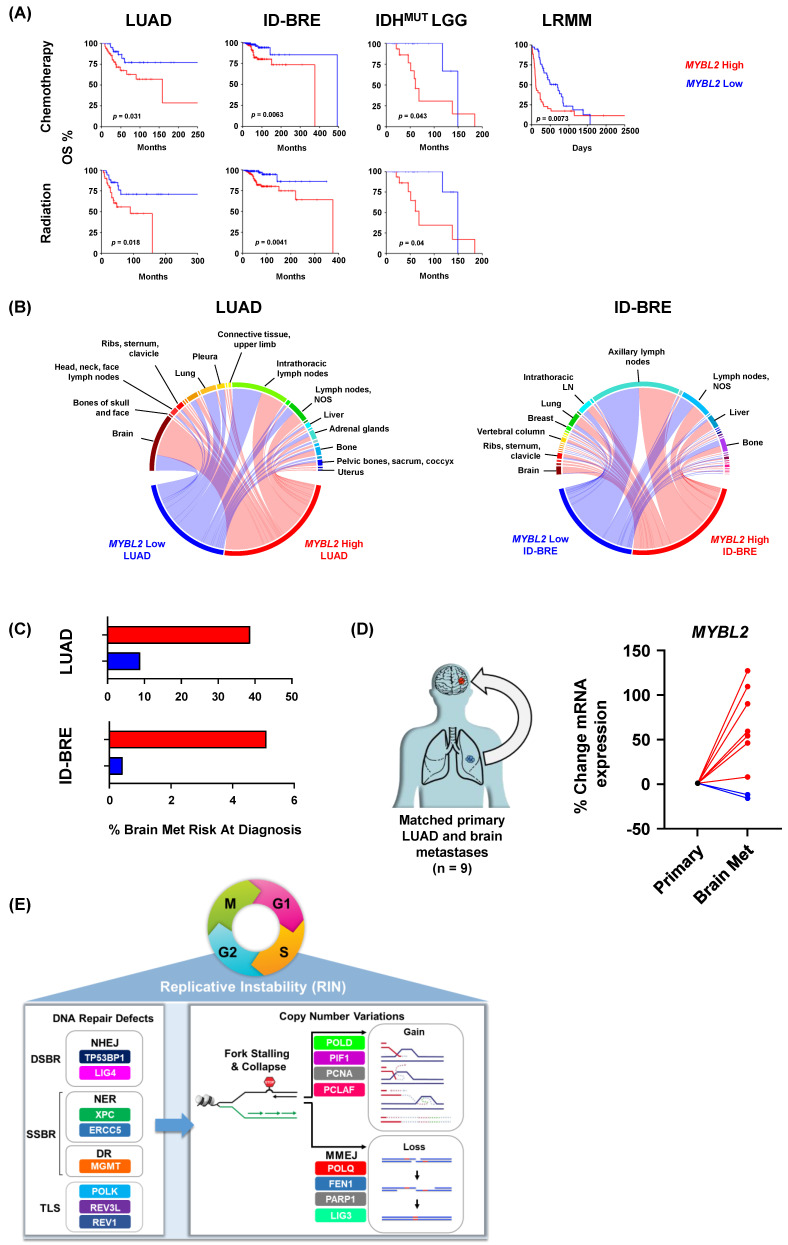
Elevated *MYBL2* expression identifies patients at risk for poor responses to therapy and distant metastases across tumor types. (**A**) *MYBL2* High patients have significantly poorer outcomes when treated with chemotherapy and irradiation regimens. Log-rank test *p*-values are displayed. (**B**) *MYBL2* High tumors metastasize to distant sites at a higher frequency, including to the brain. (**C**) *MYBL2* expression stratifies patient risk at diagnosis for brain metastasis development. LUAD: lung adenocarcinoma. ID-BRE: Invasive ductal breast cancer. IDH^MUT^ LGG: IDH-mutant lower grade glioma. LRMM: Late relapse multiple myeloma. (**D**) *MYBL2* expression is increased in brain metastases compared to patient matched primary lung adenocarcinoma tumors. (**E**) Replicative instability (RIN) accelerates genome evolution, driving cancer progression.

To test if elevated *MYBL2* expression identified patients with poor responses to therapy, we analyzed 25 tumor types provided by the Oncology Research Information Exchange Network (ORIEN). Kaplan-Meier analysis demonstrated that *MYBL2* High patients had significantly worse OS outcomes when treated with chemotherapeutics or irradiation across multiple cohorts (Figure 6A). These results fit well with our TCGA analyses where we linked elevated *MYBL2* expression with poor outcomes in treatment-naïve LUAD and IDH^MUT^ LGG (Figure 1). For ID-BRE, elevated *MYBL2* expression was not prognostic in our TCGA analysis (Supplementary Table S1). However, elevated *MYBL2* expression was highly prognostic when patients were consistently treated with chemotherapeutic or irradiation regimens. This analysis also extended our results into liquid tumors with *MYBL2* expression being robustly prognostic in the most recalcitrant form of multiple myeloma, late relapse multiple myeloma (LRMM, >4 lines of prior therapy). Analysis of COSMIC signatures confirmed resistant *MYBL2* High tumors demonstrate footprints of defective SSBR and TLS (Supplementary Figure S13). We also developed FUSED to nominate error-prone repair pathways responsible for generating genomic fusions (Methods). In *MYBL2* High samples that responded poorly to therapy, we found evidence of elevated MMEJ activity (Supplementary Figures S14–S17). These results demonstrate that DNA repair defects and increased error-prone repair potentiating *MYBL2* High disease are linked to poor responses to therapy across tumor types.

Lastly, we analyzed potential differences in metastatic dissemination (Methods). When comparing *MYBL2* High and Low cohorts, we found no difference in dissemination to sentinel lymph nodes in both LUAD (intra-thoracic lymph nodes) and ID-BRE (axillary lymph nodes) (Figure 6B). However, we found that *MYBL2* High tumors increasingly disseminated to distant sites, especially to the brain. Interestingly, we found no difference in the median time to metastasis between *MYBL2* cohorts, suggesting that observed patterns reflect tissue-specific tropisms (Supplementary Figure S18). Using combined probability, we found that *MYBL2* expression dramatically stratifies patient risk at diagnosis for developing brain metastases during their disease course (Methods, Figure 6C). These values match or exceed current genomic markers for brain metastasis risk for both LUAD and ID-BRE [51]. Analysis of primary LUAD and paired brain metastasis samples revealed that *MYBL2* expression significantly increased in 7 of 9 samples (Figure 6D). These data suggest that *MYBL2* may be a putative driver of primary carcinoma to brain dissemination.

## 4. Discussion

Across multiple tumor types, elevated *MYBL2* expression identified tumors with genomic instability, inefficient HR, and wildtype BRCA (Figure 1 and Figure 2). Analysis revealed that the genetic basis of *MYBL2* High disease is heterozygous losses of SSBR, TLS, or NHEJ effectors (Figure 3). We found that these heterozygous losses were linked to elevated replication stress, a shift in intragenic mutation position, and increased copy number alterations at RSS sites (Figure 4). Functional clustering revealed that replication stress promotes copy number alterations that rewire transcriptional programs regulating hallmarks of cancer master effectors (Figure 5). Clinically, this phenotype identifies patients at risk of poor responses to therapy (Figure 6). Our results demonstrate that patients with *MYBL2* High disease are at increased risk of distant metastases, especially to the brain (Figure 6).

In this study, we have identified a cohort of tumors characterized by replicative instability (RIN) (Figure 6E). The defining hallmark of RIN+ tumors is significant accumulation of gene-level copy number gains and losses at replication stress-sensitive genomic sites (Figure 4). Importantly, the incidence of copy number changes at these sites dramatically outnumbers somatic mutation and genomic fusion events. These findings help differentiate RIN from previously described MSI and CIN mechanisms. While we found that RIN+ tumors have significantly elevated levels of MSI (as measured by MSISensor scores, Figure 2), the overwhelming majority of RIN+ tumors fail to reach the clinically accepted “MSI-High” threshold (MSISensor > 10) [40]. It is also important to note that while RIN+ tumors have significantly higher FGA, this difference is not driven by whole-genome doublings or whole-chromosome imbalances. Furthermore, copy number changes in RIN+ tumors predominantly occur at the sub-chromosomal arm level and impact individual genes. These data clearly highlight that RIN is a distinct mechanism of genome evolution separate from CIN, where chromosome missegregation generates unstable karyotypes marked by frequent genomic translocations and chromosome number imbalances [52]. Collectively, our work supports a model where RIN accelerates genomic evolution during replication, as opposed to missegregation during mitosis, as with CIN. Genetically, we found that RIN is coincident with heterozygous losses of SSBR, TLS, and NHEJ repair effectors (Figure 3). Analysis of tissue-specific driver events revealed that RIN was not consistently linked to specific driver alterations (Figure 1). Phenotypically, we find that RIN+ tumors upregulate genes controlling the replication stress response, MMEJ, FA, and checkpoint machinery (Figure 3). It is important to note that RIN develops across cancer types. Specifically, we find that RIN develops in cancers of the lung, brain, pancreas, uterus, connective tissue, breast, and hematopoietic compartment (Figure 1 and Figure 6).

In this manuscript, we show that elevated *MYBL2* expression and RIN are intimately linked. The association of MYBL2 with RIN is both direct and indirect. In normal cells, MYBL2 is transcriptionally and post-translationally regulated by the cell cycle [53]. As cells enter S-phase, MYBL2 is transcriptionally upregulated. During S-phase, MYBL2 is phosphorylated by CCNA:CDK2 and regulates transcription. As cells progress through G2, MYBL2 upregulates FOXM1 and other effectors that promote G2/M progression. MYBL2 is then hyper-phosphorylated by CCNA:CDK2 and targeted for degradation. Given this, elevated expression of *MYBL2* mRNA is a robust marker of cells arrested prior to mitosis. In this study and our previous work, we have demonstrated that *MYBL2* expression is tightly associated with upregulation of DNA repair genes that sense replication stress [13]. This fits well when considering the mechanisms through which signaling pathways coordinate cell cycle arrest following replication stress. Upon replication stress, ATR activates CHK1 [18]. CHK1 phosphorylates CDC25 proteins and inhibits their phosphatase activity, halting cell cycle progression. By doing so, CHK1 blocks CCNB1:CDK1 activity that drives mitotic progression. Increased MYBL2 expression and activity are indirect effects of CHK1 mediated arrest. This indicates that increased MYBL2 promotes genomic evolution during replication, driving RIN. Taken together, increased *MYBL2* expression and activity are robust markers of RIN. While we find that MYBL2 expression robustly identifies patients with poor outcomes across tissue types, it should be noted that MYBL2 expression as a single marker did not stratify all known tumor types. Moving forward, it will be imperative to identify additional biomarkers that can identify this aggressive RIN phenotype that likely extends to other tumor types besides those described here (Supplementary Table S1).

Therapy resistance and metastasis directly impact patient survival. Our results indicate that RIN+ tumors respond poorly to chemotherapy and irradiation. These findings fit when considering the genetic background of these tumors. Chemotherapy and irradiation are designed to overwhelm the replicative machinery, causing cell death. Studies have demonstrated that upregulation of inter-strand crosslink repair (FA), checkpoint signaling, and error-prone repair (MMEJ) pathways confer resistance to these therapies [54,55]. Because RIN+ tumors carry heterozygous losses in SSBR, TLS, and NHEJ effectors, they experience chronic replication stress. To cope with this stress, therapy-naïve tumors upregulate FA, checkpoint, and MMEJ pathways. In doing so, these tumors become primed for resistance to DNA-damaging therapies. In addition to therapy resistance, heightened replication stress and error-prone repair promote copy number alterations in key regulators of hallmarks of cancer processes. For instance, we find that this mechanism underlies dysregulation of *TMEM173*, *JAK2*, *DAPK2*, *ERBB2*, and *NF1*, among others (Figure 5). Dysregulation of these and other effectors allow cancers to evade immune destruction, resist anoikis-driven apoptosis, achieve growth-factor independent signaling, and ultimately metastasize. Additionally, gains in *LIN9* and *LIN37* further potentiate this phenotype by increasing MYBL2 expression and activity. These alterations dramatically shorten the molecular time required for developing aggressive cancers capable of distant metastases. Consistent with this, we find that *MYBL2* High tumors are more likely to metastasize to distant sites, especially to the brain. Collectively, our results indicate that RIN is a pan-cancer driver of progression.

Our results have important implications for treatment and clinical trial designs. As RIN+ tumors respond poorly to therapy, clinical trials should explore targeted therapy combinations in the refractory setting. Given that RIN+ tumors display large quantities of neoantigens, the question of immunotherapy response is highly relevant. Because therapy-naïve RIN+ tumors exhibit highly hypoxic, MDSC-rich microenvironments, it may be unlikely that these tumors achieve durable responses to anti-PD1/PDL1. In our ORIEN cohorts, we found RIN+ tumors are associated with increased *LAG3* and *TIGIT*, despite showing no difference in *PDL1* (data not shown). This raises the possibility that anti-LAG3 and anti-TIGIT inhibitors may be better suited for treating RIN+ tumors. One of our most important discoveries is that increased *MYBL2* expression stratifies patient risk for brain metastases. While the average risk for brain metastases for all lung cancers is ~15%, we find that *MYBL2* High LUAD patients have a risk of ~40% while *MYBL2* Low have a risk of <10% (Figure 6) [51]. A similar dichotomy is observed in ID-BRE, where the reported risk for brain metastases for breast cancer patients is ~5%. Here, we find that *MYBL2* High ID-BRE risk is 5% while *MYBL2* Low ID-BRE is <1%. These results strongly argue for increased screening for brain metastases in patients with *MYBL2* High disease.

## 5. Conclusions

For decades, chromosomal instability (CIN) has been viewed as the major driver of genomic instability. Data presented in our manuscript support an additional model wherein thousands of intra-chromosomal gains and losses are driven by stalled or collapsed replication intermediates as opposed to chromosomal missegregation events. Collectively, our data demonstrate that there exists a pan-cancer class of tumors driven by replicative instability (RIN), unifying seemingly disparate tumors.

Moving forward, further study of RIN is urgently needed. Immunohistochemistry markers need to be identified and validated. New mouse models and cell line systems are required to identify therapeutic vulnerabilities that can be explored in clinical trials. Any advances in targeting RIN have the potential to drastically improve patient outcomes across tumor types.

## Data Availability

Some data analyzed in this study are subject to the following licenses/restrictions: Access to ORIEN data is controlled by M2Gen and the ORIEN consortium. Requests to access these datasets should be directed to https://www.oriencancer.org/request-an-account. Publicly available data sets were analyzed in this study. Tumor type specific survival, clinical, genomic, and hypoxia data can be found in cBioPortal (https://www.cbioportal.org/) under the following studies: Lung Adenocarcinoma (TCGA, PanCancer Atlas), Brain Lower Grade Glioma (TCGA, PanCancer Atlas), Pancreatic Adenocarcinoma (TCGA, PanCancer Atlas), Uterine Corpus Endometrial Carcinoma (TCGA, PanCancer Atlas), and Sarcoma (TCGA, PanCancer Atlas). Mutation Position Scores were generated using TCGA MAF file mc3.v0.2.8.PUBLIC.maf.gz (gdc.cancer.gov/about-data/publications/pancanatlas). Copy number analyses for MiDAS and ERFS genes were conducted using SCNV gene level, GITSTIC 2 thresholded files for LUAD, LGG, PAAD, UCEC, and SARC studies (linkedomics.org). ConsensusTME scores were generated using RSEM gene normalized RNA-seq files downloaded from GDAC FireBrowse (firebrowse.org) for each tumor study. Neoantigen and pMHC data are available from Thorsson et al. (Supplementary files: TCGA_PCA.mc3.v0.2.8.CONTROLLED.filtered.sample_neoantigens.10062017.tsv, TCGA_pMHC_SNV_sampleSummary_MC3_v0.2.8.CONTROLLED.170404.tsv, gdc.cancer.gov/about-data/publications/panimmune). MDSC infiltration, tumor dysfunction, and tumor exclusion scores were downloaded from the TIDE: Tumor Immune Dysfunction and Exclusion database (tide.dfci.harvard.edu/download). Genomic data and DNA repair metrics are available from Knijenburg et al. (Supplementary file “TCGA_DDR_Data_Resources.xlsx”).

## Supplementary Materials

The following are available online at https://www.mdpi.com/article/10.3390/biom12111570/s1, Figure S1: *MYBL2* High lung adenocarcinoma replication stress sensitive site labeled functional cluster analysis; Figure S2: *MYBL2* High IDH-mutant lower grade glioma replication stress sensitive site labeled functional cluster analysis; Figure S3: *MYBL2* High pancreatic adenocarcinoma replication stress sensitive site labeled functional cluster analysis; Figure S4: *MYBL2* High endometrial carcinoma replication stress sensitive site labeled functional cluster analysis; Figure S5: *MYBL2* High sarcoma replication stress sensitive site labeled functional cluster analysis; Figure S6: *MYBL2* High tumors exhibit uniquely dysregulated tumor microenvironments; Figure S7: Lung adenocarcinoma ConsensusTME and TIDE analysis; Figure S8: IDH-mutant lower grade glioma ConsensusTME and TIDE analysis; Figure S9: Pancreatic adenocarcinoma ConsensusTME and TIDE analysis; Figure S10: Endometrial carcinoma ConsensusTME and TIDE analysis; Figure S11: Sarcoma ConsensusTME and TIDE analysis; Figure S12: Hypoxia score analysis; Figure S13: ORIEN COSMIC SBS 3.2 analysis; Figure S14: ORIEN LUAD *MYBL2* High Low therapy FUSED analysis; Figure S15: ORIEN ID-BRE *MYBL2* High Low therapy FUSED analysis; Figure S16: ORIEN IDH^MUT^ LGG *MYBL2* High Low therapy FUSED analysis; Figure S17: ORIEN LRMM *MYBL2* High Low therapy FUSED analysis; Figure S18: ORIEN time to metastasis swimmer plots; Table S1: Excel sheet detailing log-rank *p* values for *MYBL2* High vs. *MYBL2* Low OS, DSS, PFS outcomes across tumor types; Table S2: Excel sheet containing final *MYBL2* High and Low patient identifiers and clinical data for all five tumor types; Table S3: Excel sheet containing WE pathway score data for *MYBL2* High vs *MYBL2* Low tumors; Table S4: Excel sheet containing replication stress score genes; Table S5: Excel sheet containing replication stress sensitive site function cluster annotation; Table S6: Excel sheet containing final replication stress sensitive site copy number alteration percentages along with RNA-seq differential expression values for all tumor cohorts; Table S7: Excel sheet containing patient cohort numbers for TCGA and ORIEN Kaplan-Meier survival analyses in Figure 1 and Figure 6.

## Abbreviations

RIN	replicative instability
MYBL2	Myb proto-oncogene like 2
TCGA	The Cancer Genome Atlas
OS	overall survival
PFS	progression free survival
DSS	disease-specific survival
LUAD	lung adenocarcinoma
IDH^MUT^ LGG	isocitrate dehydrogenase-mutant lower grade glioma
PAAD	pancreatic adenocarcinoma
UCEC	uterine corpus endometrial carcinoma
SARC	sarcoma
FGA	fraction of the genome altered
MSI	microsatellite instability
HR	homologous recombination
RPS	repair proficiency score
Combined-HRD score	combined homologous recombination deficiency score
WE score	weighted expression score
SSBR	single-strand break repair
DSBR	double-strand break repair
TLS	translesion synthesis
NER	nucleotide excision repair
NHEJ	non-homologous end-joining
DR	direct reversal repair
COSMIC	Catalog of Somatic Mutations in Cancer
SBS	single-base substitution
RS score	replication stress score
MPS	mutational position score
ERFS	early replicating fragile sites
MiDAS	mitotic DNA synthesis sites
RSS sites	replication stress sensitive sites
MDSC	myeloid derived suppressor cell
ORIEN	Oncology Research Information Exchange Network
ID-BRE	invasive ductal breast cancer
LRMM	late-relapse multiple myeloma
FUSED	FUSion Error-prone repair Detection
CIN	chromosomal instability
MMEJ	microhomology mediated end-joining
FA	Fanconi Anemia
GO	Gene Ontology
