# Supplementary material for: Replicative Instability Drives Cancer Progression
Source: Biomolecules. 2022 Oct 26;12(11):1570. doi: 10.3390/biom12111570 (PMC9688014; doi:10.3390/biom12111570)
Supplement: Supplementary file 1 [file biomolecules-12-01570-s001.zip › biomolecules-1894415-supplementary.pdf]

Supplementary Figures

Morris et al.
Supp. Figure 1

LUAD

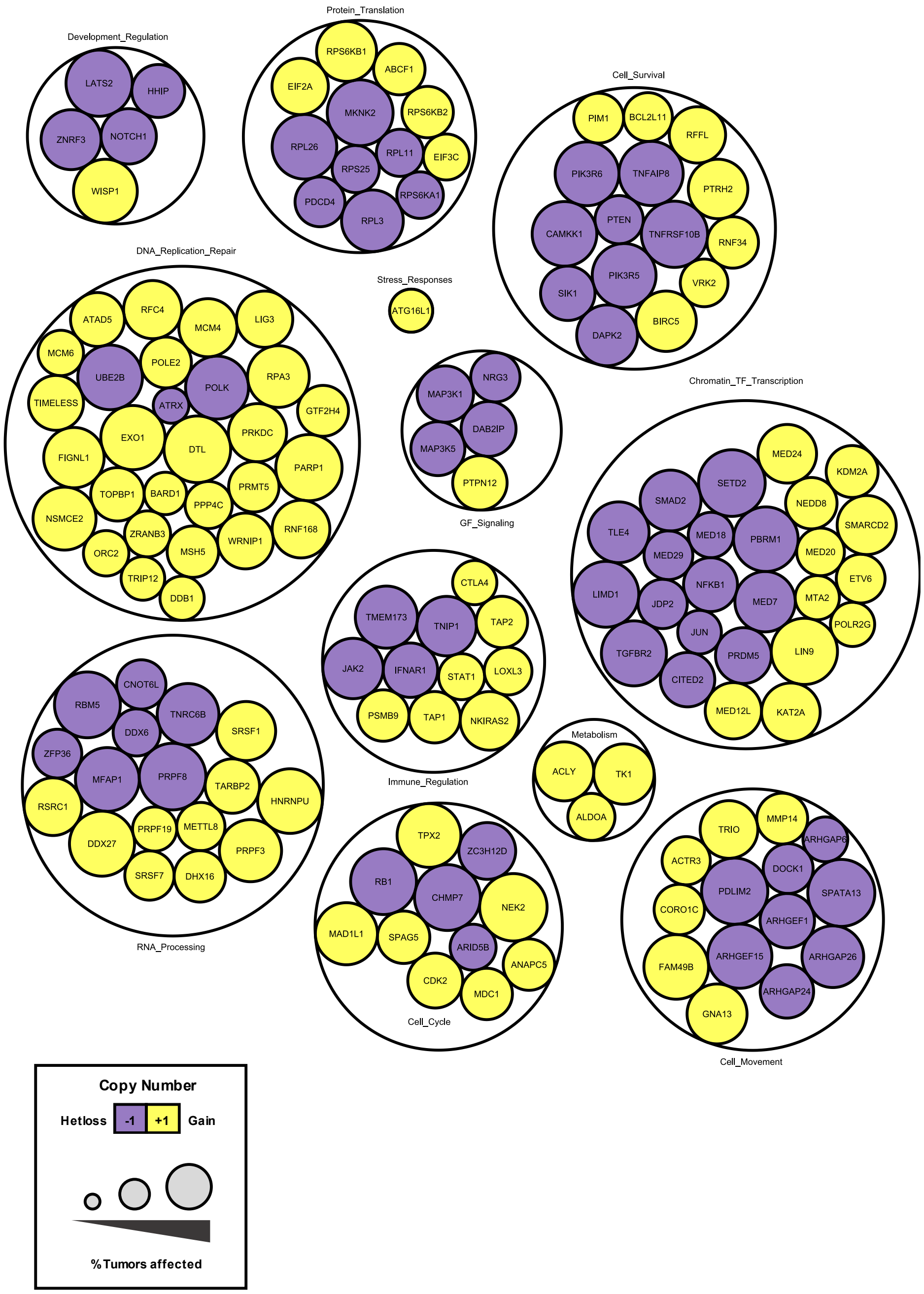

Figure_S1: *MYBL2* High lung adenocarcinoma replication stress sensitive site labeled functional cluster analysis.

Morris et al.
Supp. Figure 2

IDH^{MUT} LGG

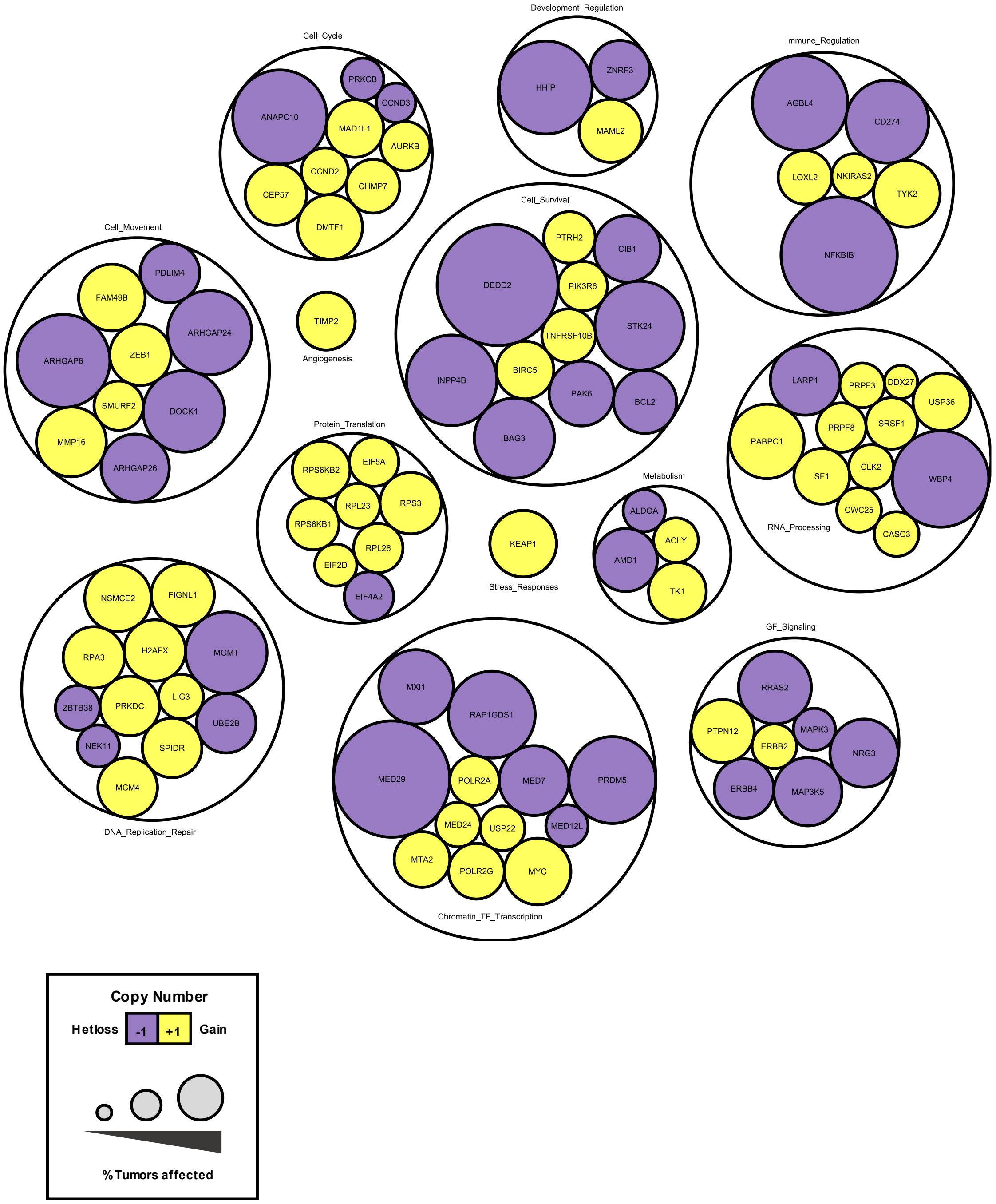

Figure_S2: ***MYBL2*** **High IDH-mutant lower grade glioma replication stress sensitive site labeled functional cluster analysis.**

Morris et al.
Supp. Figure 3

PAAD

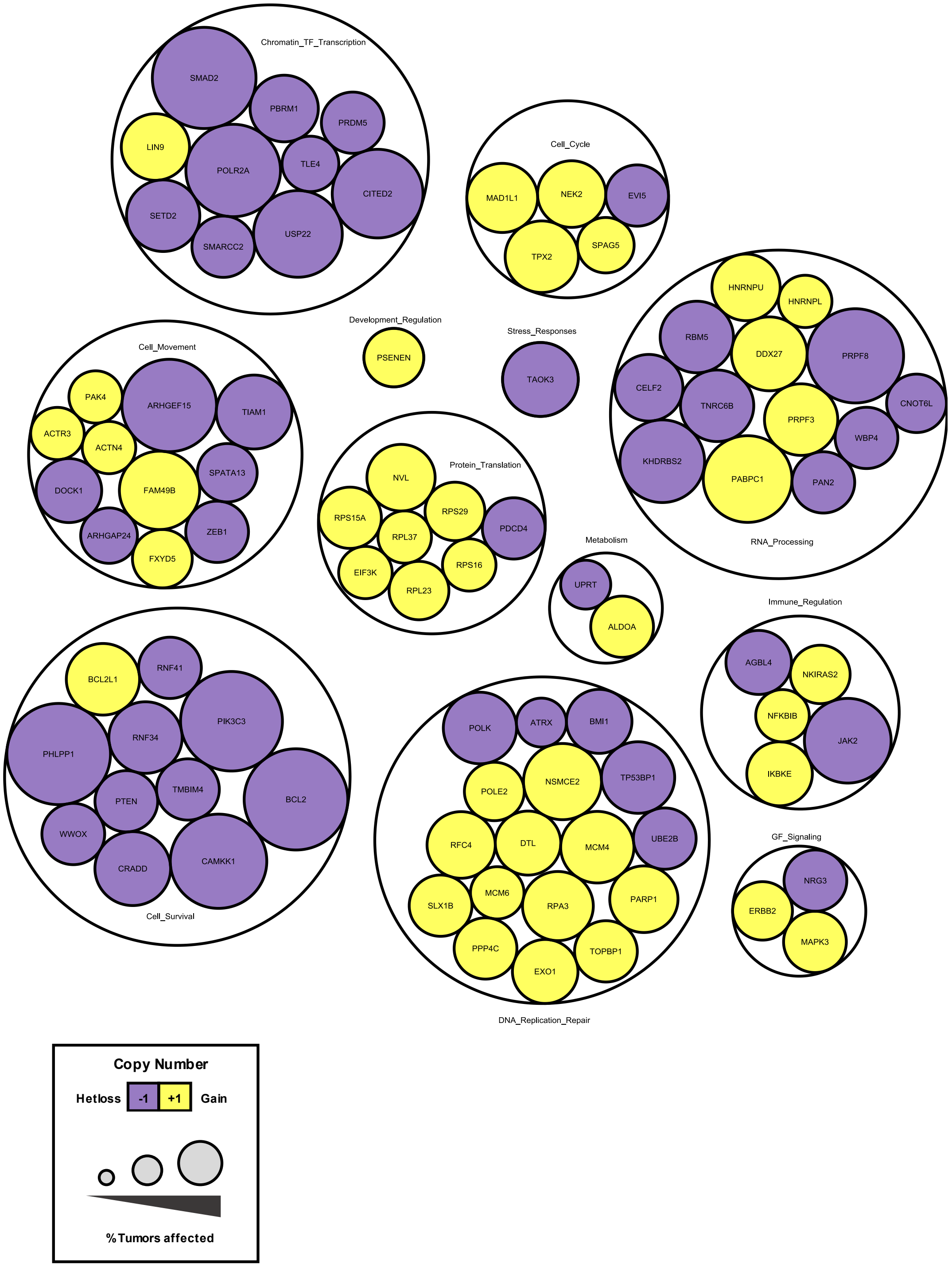

Figure_S3: *MYBL2* High pancreatic adenocarcinoma replication stress sensitive site labeled functional cluster analysis.

Morris et al.
Supp. Figure 4

UCEC

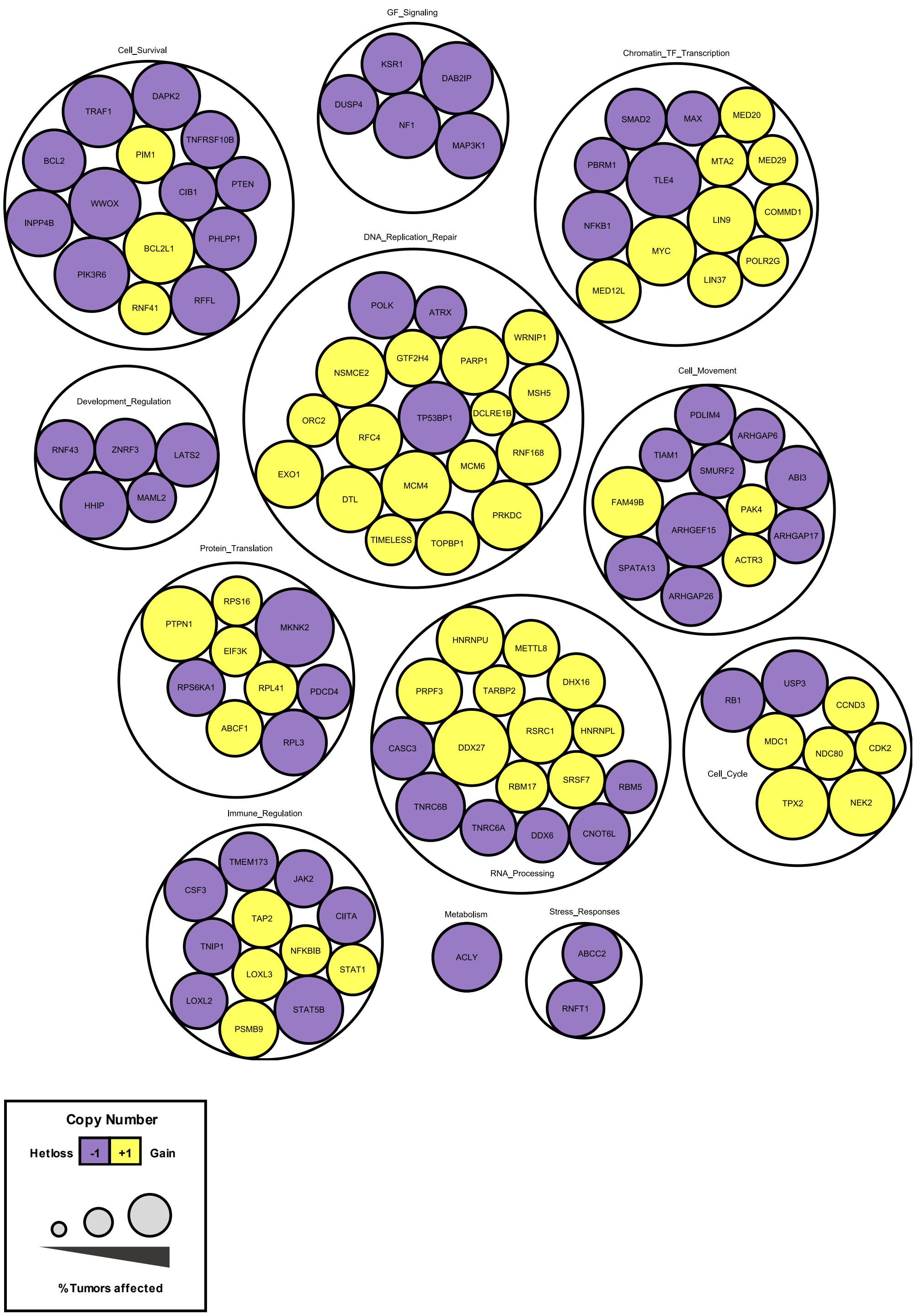

Figure_S4: *MYBL2* High endometrial carcinoma replication stress sensitive site labeled functional cluster analysis.

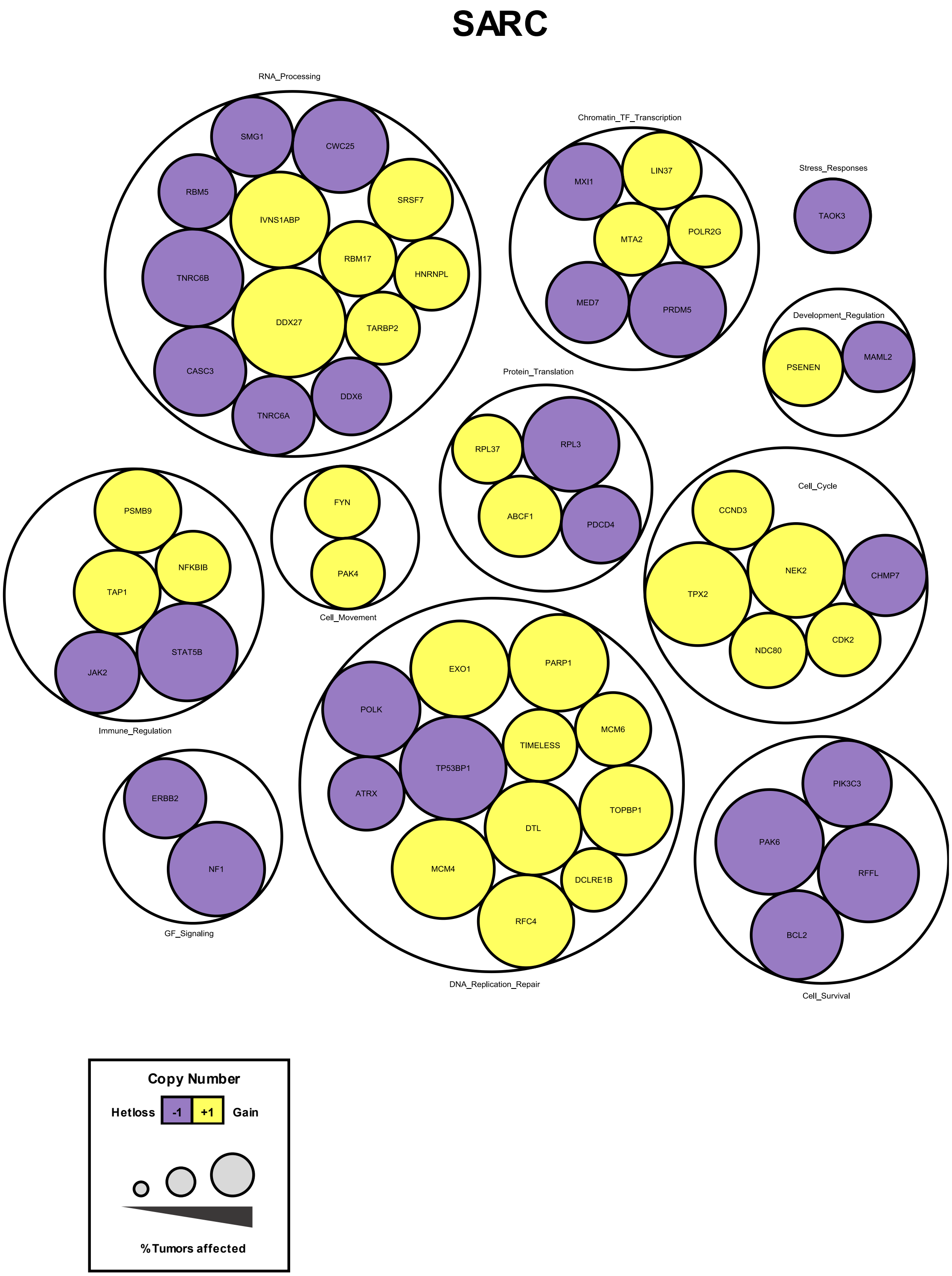

Figure_S5: *MYBL2* High sarcoma replication stress sensitive site labeled functional cluster analysis.

Morris et al.
Supp. Figure 6

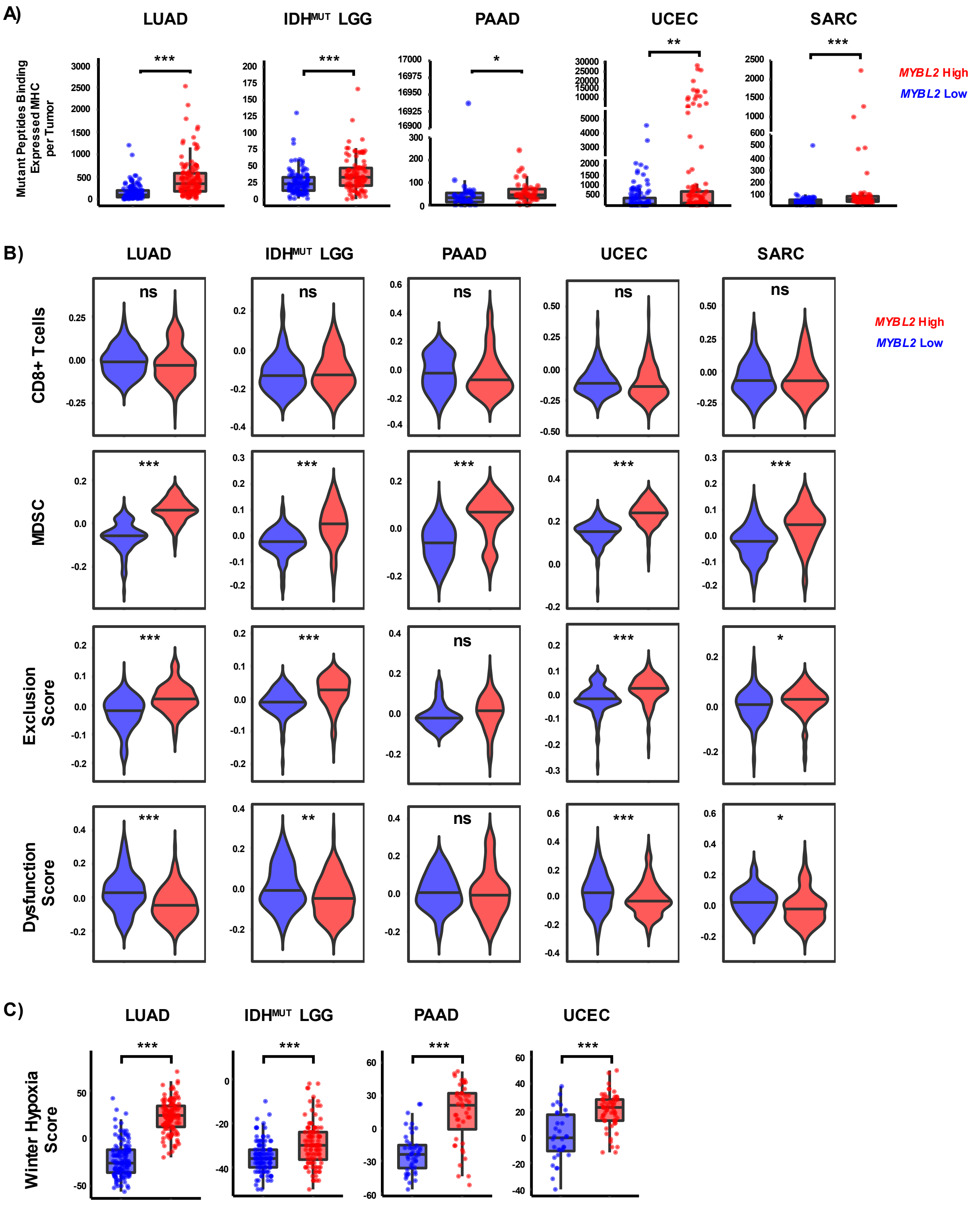

Figure_S6: *MYBL2* High tumors exhibit uniquely dysregulated tumor microenvironments. A) *MYBL2* High tumors contain significantly greater numbers of mutant peptides that bind to patient-matched, expressed, pMHC complexes. **B)** Immune infiltration estimation algorithms indicate that *MYBL2* High tumors are significantly more immunosuppressive. **C)** *MYBL2* High tumors are highly hypoxic. **A), B), C)** Wilcoxon, $p < 0.05$, *; $p < 0.01$, **; $p < 0.001$, ***.

Morris et al.
Supp. Figure 7

LUAD

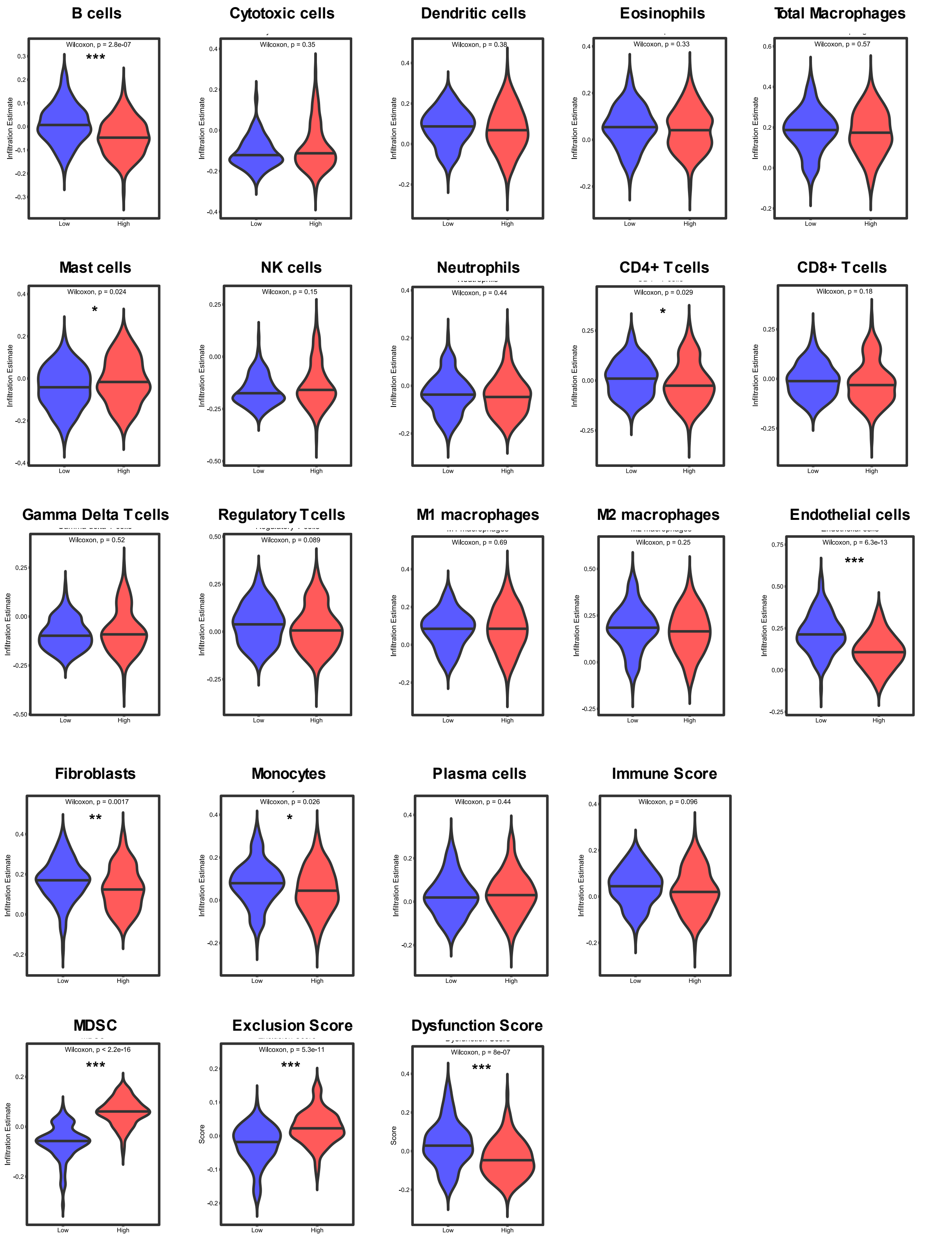

Figure_S7: Lung adenocarcinoma ConsensusTME and TIDE analysis.

Morris et al.
Supp. Figure 8

IDH^{MUT} LGG

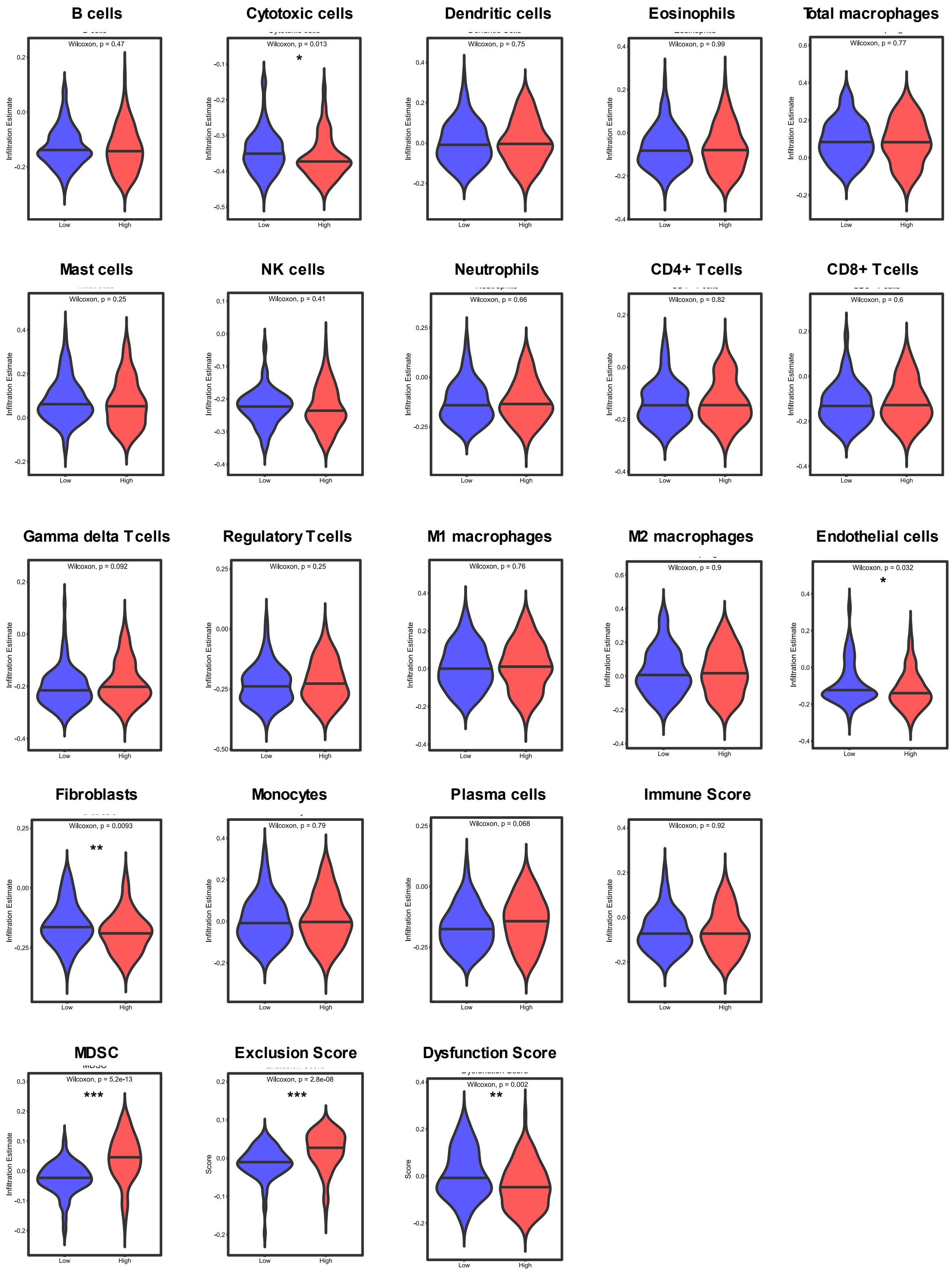

Figure_S8: IDH-mutant lower grade glioma ConsensusTME and TIDE analysis.

Morris et al.
Supp. Figure 9

PAAD

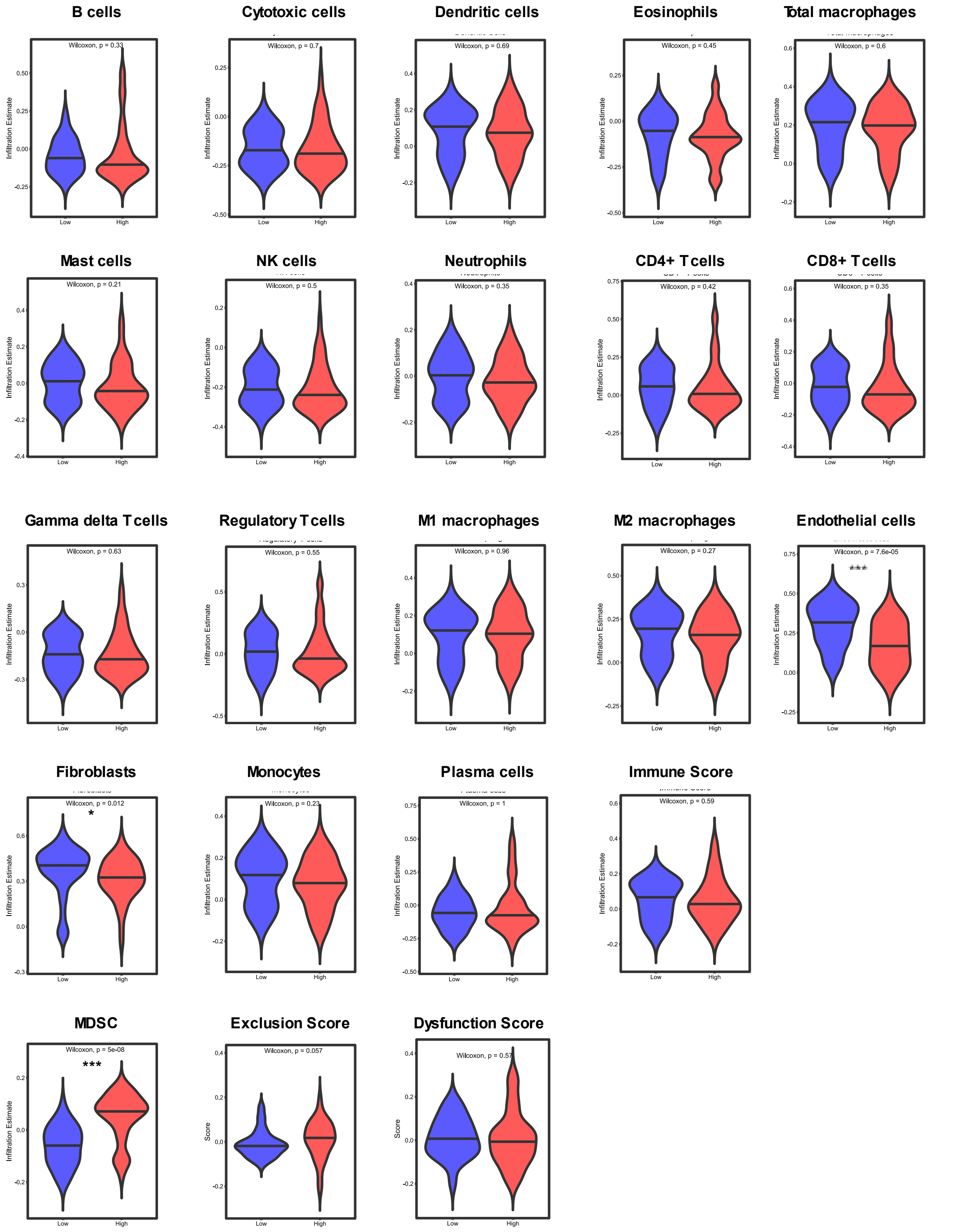

Figure_S9: Pancreatic adenocarcinoma ConsensusTME and TIDE analysis.

Morris et al.
Supp. Figure 10

UCEC

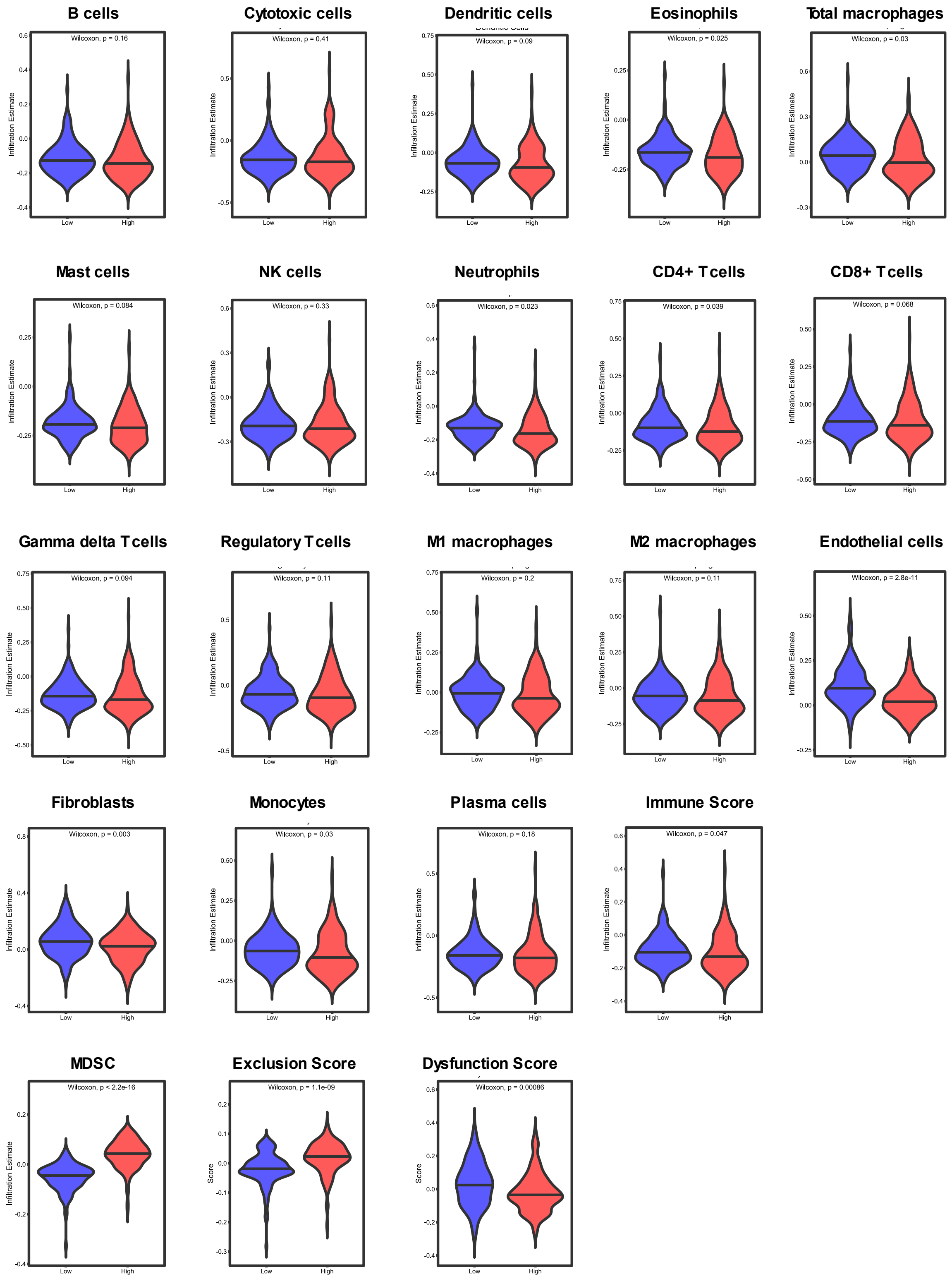

Figure_S10: Endometrial carcinoma ConsensusTME and TIDE analysis.

Morris et al.
Supp. Figure 11

SARC

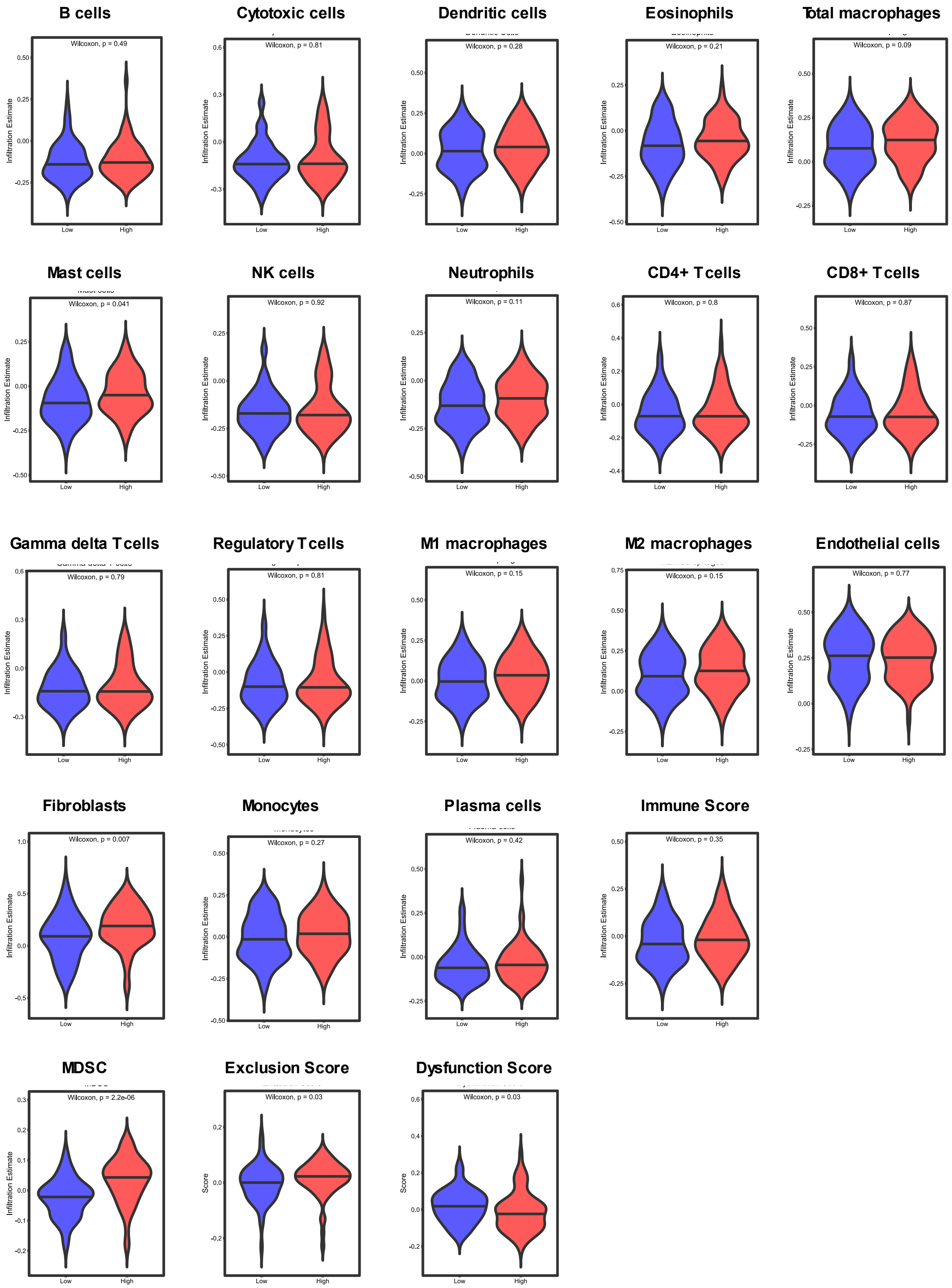

Figure_S11: Sarcoma ConsensusTME and TIDE analysis.

Morris et al.
Supp. Figure 12

LUAD

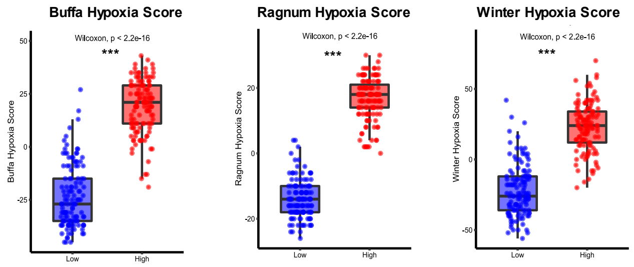

IDH^{MUT} LGG

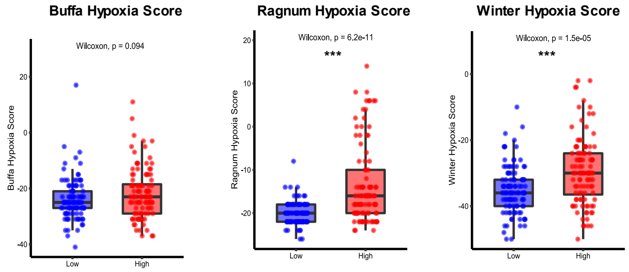

PAAD

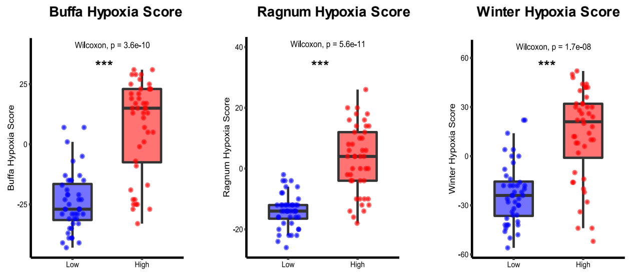

UCEC

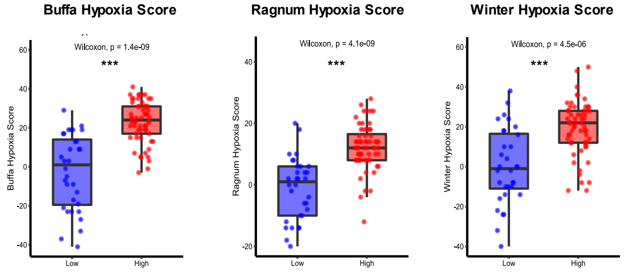

SARC

Buffa Hypoxia Score **Ragnum Hypoxia Score** **Winter Hypoxia Score**

Hypoxia scores not available for SARC patients

Figure_S12: Hypoxia score analysis.

Morris et al.
Supp. Figure 13

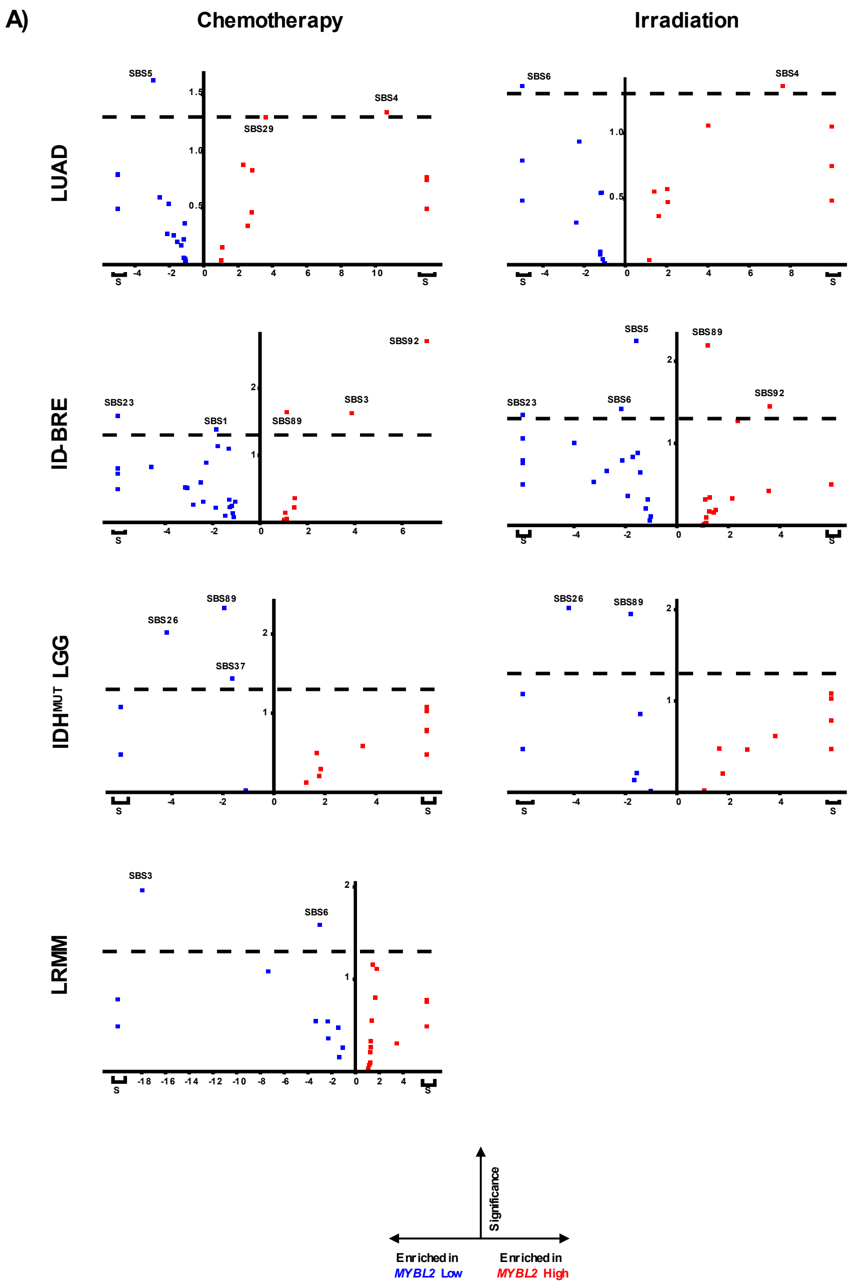

Figure_S13: ORIEN COSMIC SBS 3.2 analysis.

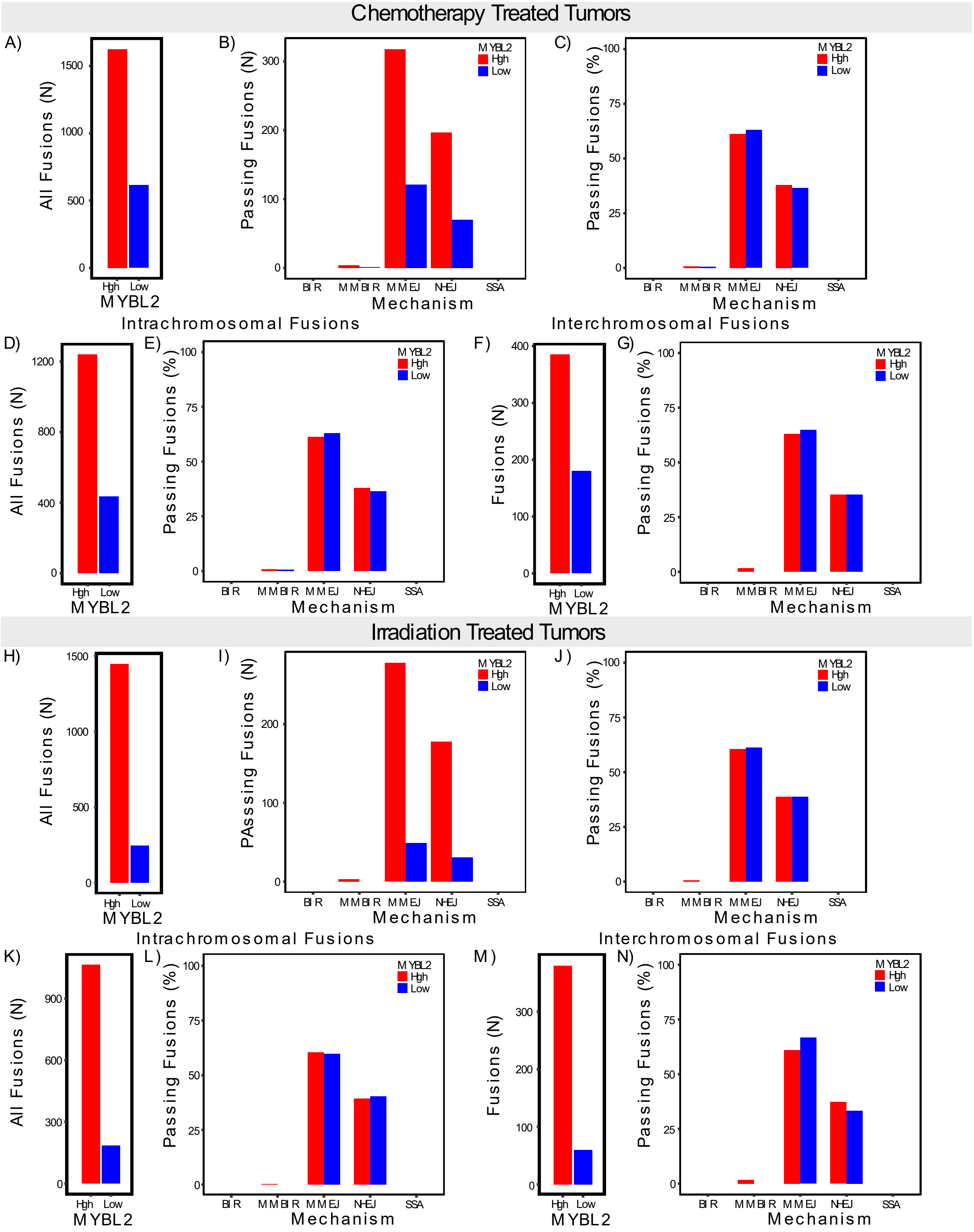

Figure_S14: ORIEN LUAD *MYBL2* High Low therapy FUSED analysis.

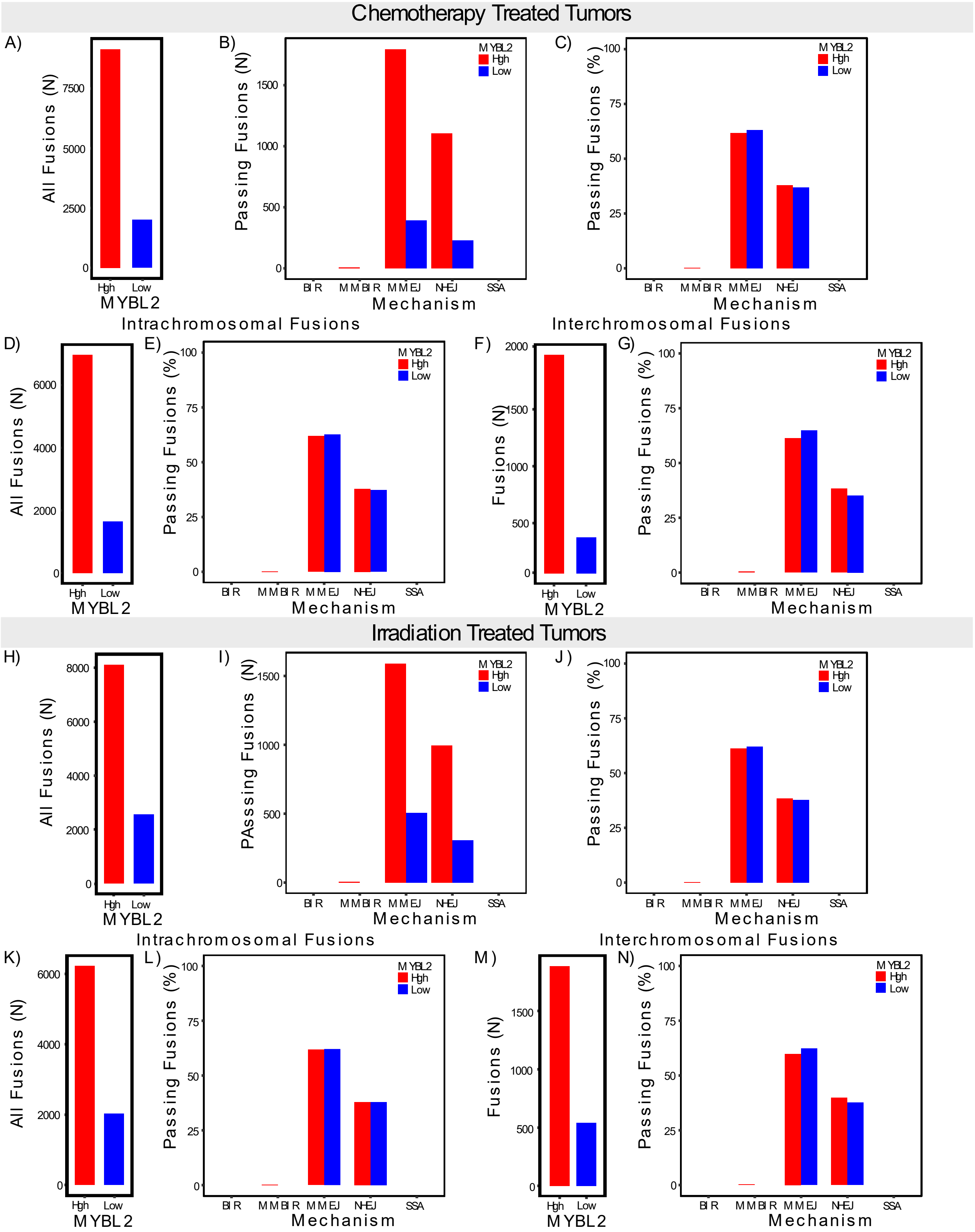

Figure_S15: ORIEN ID-BRE *MYBL2* High Low therapy FUSED analysis.

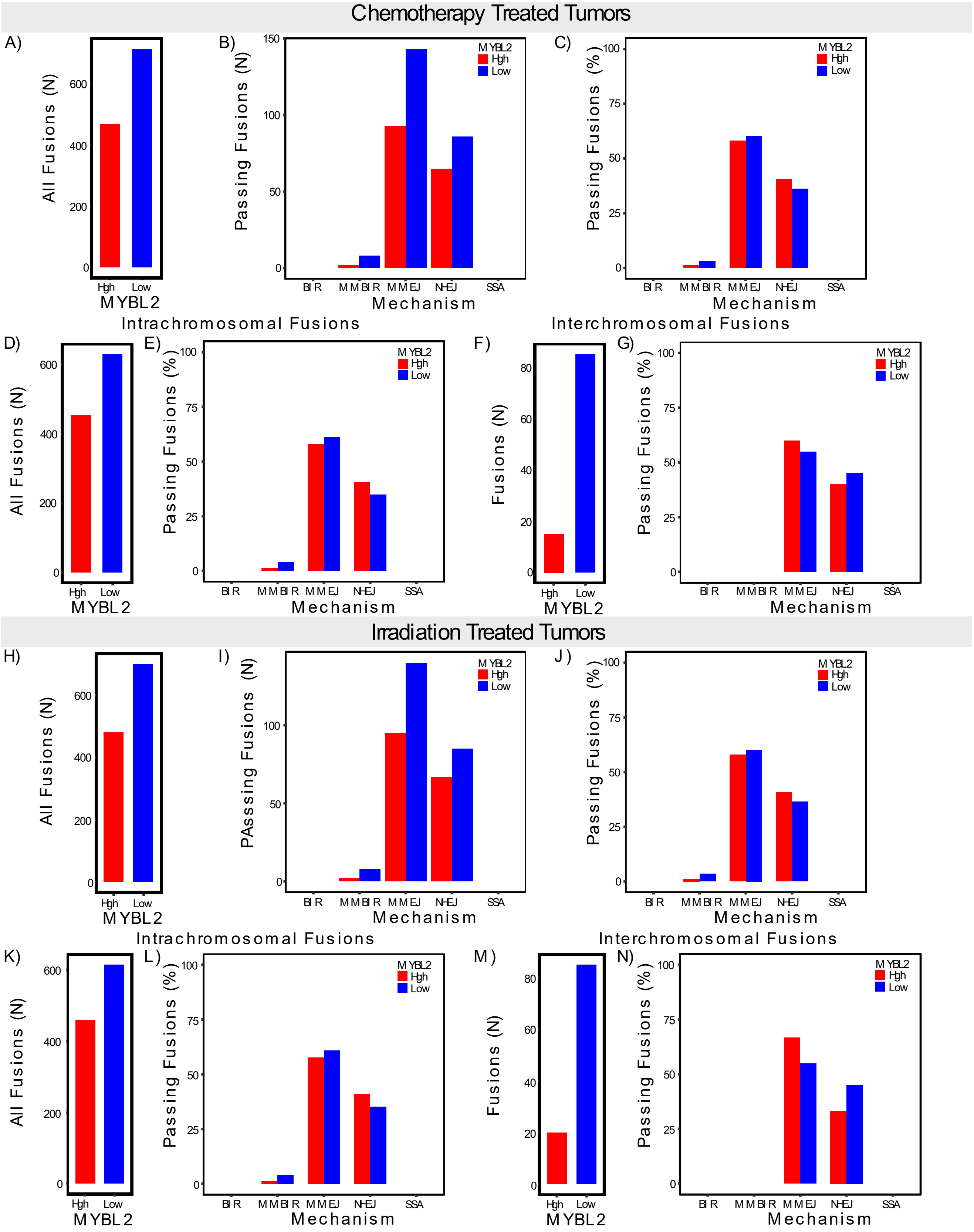

Figure_S16: ORIEN IDH^{MUT} LGG *MYBL2* High Low therapy FUSED analysis.

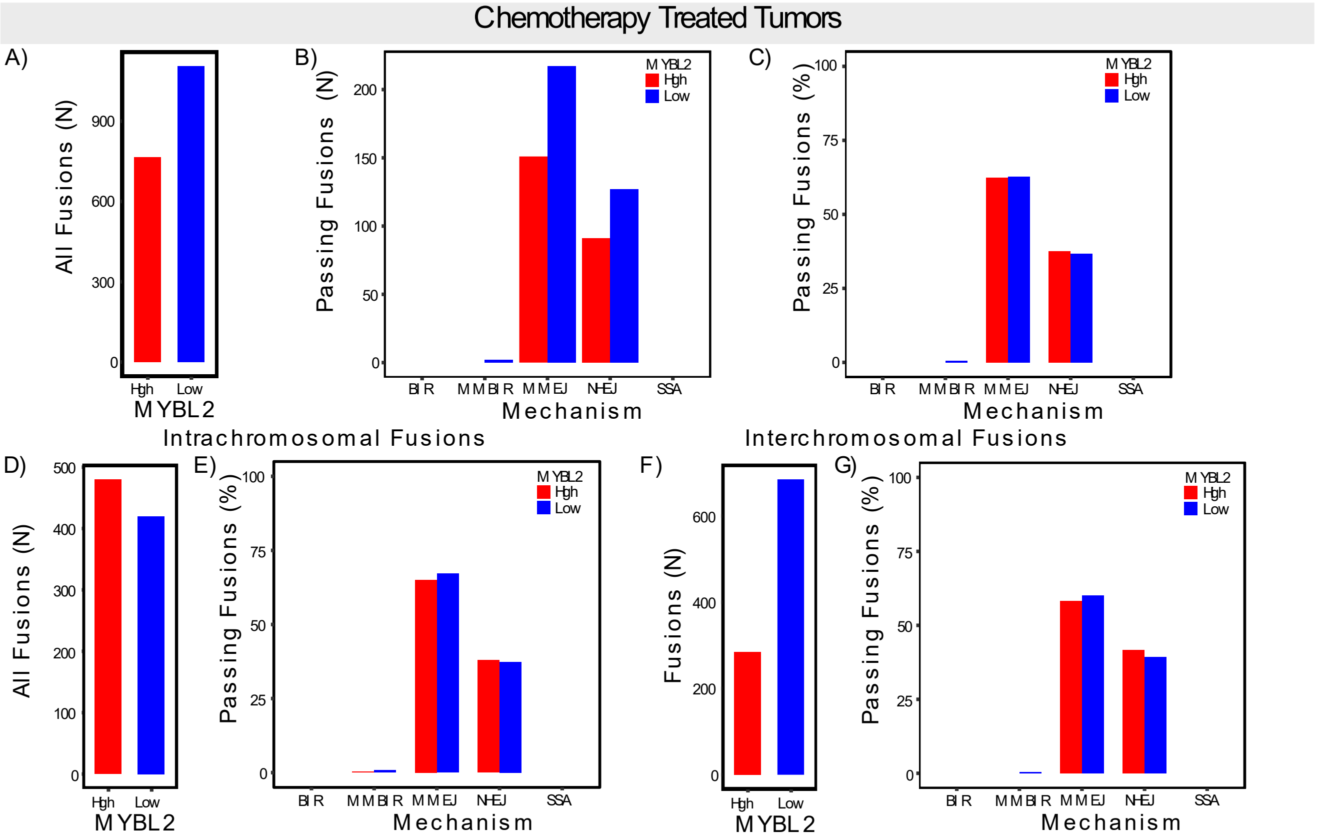

Figure_S17: ORIEN LRMM *MYBL2* High Low therapy FUSED analysis.

Morris et al.
Supp. Figure 18

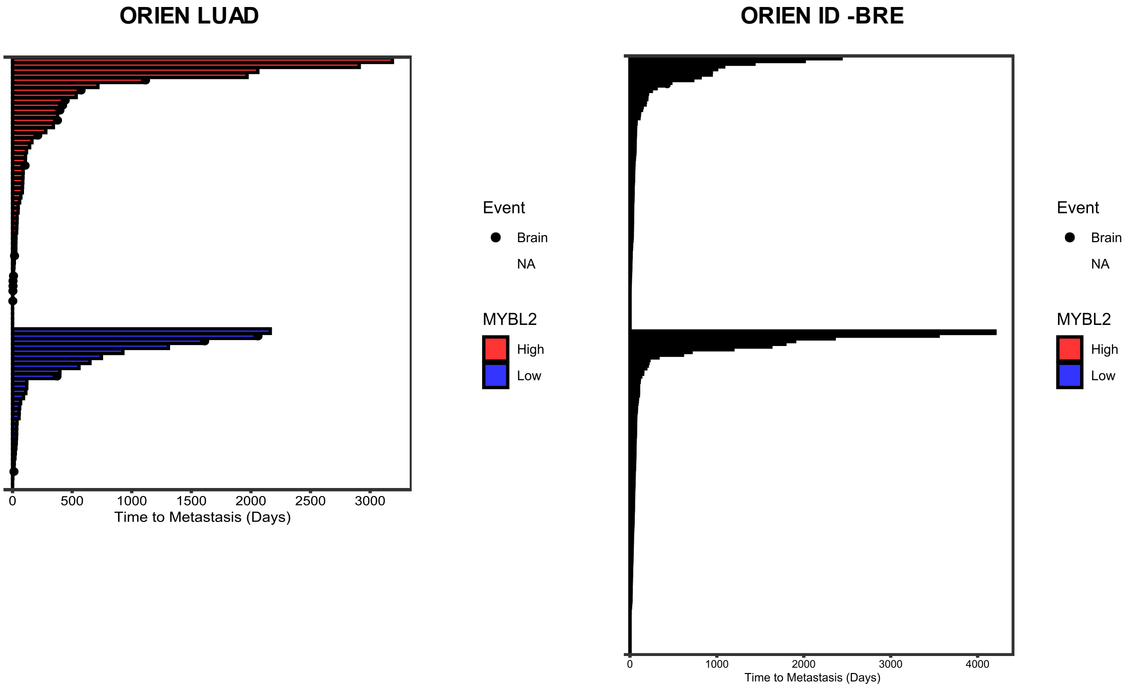

Figure_S18: ORIEN time to metastasis swimmer plots.
